# Image encryption with 6D hyperchaotic system and vision transformer autoencoder

**DOI:** 10.1038/s41598-025-34378-5

**Published:** 2026-01-14

**Authors:** Alenrex Maity, Yen-Lin Chen, Wassim Alexan, Por Lip Yee, Bibhas Chandra Dhara

**Affiliations:** 1 Department of Information Technology, Jadavpur University, Kolkata, India; 2 Department of Computer Science and Information Engineering, National Taipei University of Technology, Taipei, 106344 Taiwan; 3 Communications Department, Faculty of Information Engineering and Technology, German University in Cairo (GUC), Cairo, Egypt; 4 Center of Research for Cyber Security and Network (CSNET), Faculty of Computer Science and Information Technology, Universiti Malaya, Kuala Lumpur, 50603 Wilayah Persekutuan Malaysia

**Keywords:** Information security, Hyperchaotic system, ViT autoencoder, Color image encryption, Trifid Cipher, Engineering, Mathematics and computing

## Abstract

This study proposes a fast image encryption method for color images, integrating an autoencoder to compress the image and a 6D hyperchaotic system to ensure enhanced security. Initially, a hash value is obtained from the original color image. The hash value, which serves as the secret key of the proposed encryption method, is used to initialize the state variables of the hyperchaotic system, which produces six distinct pseudo-random sequences. The input image is then compressed into a latent image (lossy) using a Vision Transformer Autoencoder model. This latent image is scrambled using chaotic sequences and a Random Shuffle technique. Diffusion is achieved through the Trifid Cipher transformation, which utilizes the remaining chaotic sequences to manipulate pixel values, thereby yielding a cipher version of the latent image. The suggested technique is faster and significantly enhances security compared to the state-of-the-art methods. This method achieves an average entropy of 7.9986, a correlation coefficient close to zero $\approx$ 0.00004, and key sensitivity analysis gives NPCR = 99.6110% and UACI = 33.4637%. Moreover, the key space of $2^{512}$ confirms that the proposed scheme offers strong resistance against brute-force attacks.

## Introduction

The phenomenal growth of Internet technologies and the ever-increasing exchange of multimedia data have raised the alarm regarding the security of digital images^1^. Since digital images are the most widely used form of multimedia today, their proliferation through various platforms, including but not limited to social networks, telemedicine systems, and IoT networks, puts them at risk^2,3^. With the large volume of information available on public networks, there are more possibilities for such networks to be illegally accessed, altered, or breached. Therefore, data security, particularly effective measures against unauthorized access or alterations of images, has emerged as an urgent issue due to these dangers.

In high-stakes applications like medical imaging, online banking, and secure communications, the protection of digital images is paramount^4^. The leakage of sensitive information, whether personal photographs, medical documents, or proprietary business designs, can lead to serious consequences, including financial losses and compromised privacy. These images are very sensitive, and if accessed by unauthorized persons, they not only jeopardize individuals but also present serious security risks to organizations. Due to the openness that characterizes the digital image, including the ability to copy, manipulate, and disseminate it, it is necessary to use high-end encryption and security for transmitting the images.

Although encryption is a well-known technique used for data protection, the application of classical cryptosystems, such as DES^5^, AES^6^, and RSA^7^, is insufficient for image encryption. These systems, initially developed for text, fail to effectively address the essential characteristics of digital images, such as high data redundancy and strong inter-pixel correlations. Their processing requirements may be suboptimal, especially for massive image data^8^.

Subsequently, new chaos-based encryption techniques have been developed, which provide higher security and faster data processing. Chaos is characterized by nonlinear dynamics, sensitivity to initial conditions, unpredictability, randomness, and ergodicity, all of which make it well-suited for cryptographic applications^9^. Due to these properties, the chaos-based encryption technique has garnered considerable attention in recent years^10^. Dispersive maps employed in encryption systems are typically classified as one-dimensional (1D) and multidimensional types. Only a few initial values and the control parameters are needed for the 1D maps; however, their efficiency is reduced if the parameters are distributed within definite intervals, making the generated sequence susceptible to extraction. These issues have since been rectified by the introduction of multi-dimensional maps^11,12^, which employ trigonometric and polynomial functions that make the maps computationally more random and unpredictable, thus providing higher security to the images being encrypted.

The substitution box (S-Box) is an important component in classical techniques such as DES and AES, as it performs a non-linear operation that makes cryptanalysis more difficult due to the exponential increase in possible alternatives. In some recent image encryption methods, S-Box is defined using elliptic curve, chaotic sequences, or geometrical features to achieve more non-linearity and dynamism. In^13^, S-Boxes are designed using the Mordell elliptic curve over GF($2^n$), with different *n* values like 8, 9, 10, 11, 12, etc. The use of an elliptic curve gives more dynamism and nonlinearity in the S-Boxes and also achieves high security using a smaller key. In their research work, Ahmad *et al.*^14^ present a new method to define a 3D S-Box. The Lorenz chaotic system is employed to generate streams of random numbers, which are then used to compute the S-Box. The work^15^ introduces a novel S-Box design that merges octagonal geometry with chaotic dynamics to enhance security. The mathematical construct of an octagon provides the necessary confusion in the S-Box. This structure improves the nonlinearity of the S-Box.

In modern encryption, image compression is essential to handle high-resolution color images. Image compression reduces image size, resulting in lower storage requirements, reduced transmission latency, and lower computational overhead. It makes the image ideal for resource-constrained environments, such as IoT systems, telemedicine, surveillance, and remote sensing, where processing power and transmission bandwidth are limited^2^. Traditional compression techniques may cause the loss of important semantic details and often fail to preserve the structural integrity required for secure and accurate image reconstruction.

Deep learning-based autoencoders^16^ have become popular due to their ability to perform learned, task-specific compression, thus overcoming these constraints. Although autoencoder-based compression is inherently lossy, it offers several significant benefits over traditional methods. Compact latent representations are learned using autoencoders, which eliminate unnecessary information while preserving the most significant features of the image. This enables adequate reduction of dimensionality, resulting in low memory usage and accelerated encryption and decryption. Due to these characteristics, autoencoders are particularly well-suited for applications where secure handling and real-time performance are essential, and approximate reconstruction is acceptable.

Vision Transformers (ViT) based autoencoders have recently become a powerful extension of this foundation^17–19^. Compared to conventional convolutional autoencoders, which typically focus on local features, ViTs can better capture global contextual patterns and long-range dependencies by utilizing self-attention techniques. ViTs enable the scalable and adaptable handling of high-resolution images by tokenizing them into fixed-size patches and processing them as a series of tokens. This global representation improves compression quality and enhances the subsequent encryption process by providing more compact and informative features.

Our study presents a color image encryption method that integrates a 6D hyperchaotic system, a ViT-based autoencoder, a random shuffle technique, and the Trifid cipher. The SHA3-512 algorithm is first applied to the original color image to produce a 512-bit hash value (*H*), which is used as the secret key for the proposed encryption method. This hash value is then used to set up the state variables of the 6D hyperchaotic system. The hyperchaotic system subsequently generates six pseudo-random sequences $\{X_1, X_2,..., X_6\}$. The original image is then passed through a Vision Transformer Autoencoder (ViTAE) model, where the encoder component extracts a latent representation of the image. The latent image undergoes a multistage encryption process. First, it is scrambled with the help of the chaotic sequence $X_1$. Subsequently, a Random Shuffle technique is applied using $X_2$ to disrupt the pixel positions further. Following the scrambling phase, the latent image is diffused using the Trifid Cipher transformation utilizing $\{X_3, X_4, X_5, X_6\}$, resulting in the encrypted latent image. The key contributions of this study can be outlined as follows:

- A new 6D hyperchaotic system is proposed, with a comprehensive analysis of its dynamic properties.
- An efficient color image encryption strategy is developed by integrating a Vision Transformer (ViT) autoencoder with a 6D hyperchaotic system, a random shuffle technique, and the Trifid cipher.
- Encrypts the latent image extracted by the ViTAE encoder, rather than the entire image, for efficiency and to make it suitable for resource-constrained environments.
- A comprehensive evaluation and comparison with cutting-edge techniques are performed to validate the security, efficiency, and robustness of the proposed method.

The remainder of this article is outlined as follows: Section Related Works provides an overview of existing image encryption techniques. In Section 6D Hyperchaotic System, the proposed 6D hyperchaotic system is introduced, together with a detailed analysis of its dynamic properties. Section Vision Transformer Autoencoder demonstrates an overview of the ViT Autoencoder architecture, highlighting its design and functionality in image compression. Section Proposed Image Encryption Method represents the proposed encryption scheme, including the Random Shuffle technique and the Trifid Cipher transformation. Section Experimental Results and Analysis details the experimental findings and evaluates the performance of the proposed method. Lastly, Section Conclusions and Future Work provides a summary and concluding remarks of the paper.

## Related works

This section provides a summary of the state-of-the-art (SoA) image encryption techniques that utilize deep learning approaches.

Maniyath *et al.*^20^ proposed an image encryption framework combining chaotic maps and deep learning techniques. Using stacked autoencoders and feed-forward backpropagation, the system generates secure secret keys efficiently while bypassing iterative training. Fang *et al.*^21^ presented an encryption scheme by combining DCGANs, quaternions, and an improved Feistel network. Integrated with DCGANs, the hyperchaotic system generates a highly random and complex key stream. This stream and a quaternion representation are used in an enhanced Feistel network to encrypt the images. Ding *et al.*^22^ presented an encryption technique for medical images using Cycle-GAN. This model encrypts images by converting them into a hidden domain and decrypts them through image reconstruction. An ROI-mining network enables analysis by extracting key regions from encrypted images. Man *et al.*^23^ presented an encryption technique by combining CNNs with dynamic adaptive diffusion. The dual-channel design merges optical and digital encryption to enhance parallelism and reliability. Chaotic maps secure key generation, while CNN-driven chaotic sequences ensure effective scrambling. A novel image fusion technique and adaptive diffusion further strengthen security. Wu *et al.*^24^ suggested an encryption technique that integrates GAN and the Logistic map. GAN generates an intermediate image, which is encrypted using XOR with a Logistic map. Fang *et al.*^25^ suggested a block-wise image encryption scheme that employs GANs, a 1D chaotic system, and DNA encoding. In this approach, two keystreams are generated and used within a Feistel network, where encryption is performed by multiplying a block matrix. Man *et al.*^26^ developed a GAN-based random number generator trained on chaotic sequences to enhance encryption key security. They introduced selective scrambling focused on important pixel regions and edges and a superposition diffusion method using pixel blocks for more efficient diffusion. Bao *et al.*^27^ proposed an Image Scrambling Adversarial Autoencoder for secure image transmission. A CycleGAN-based encoder scrambles the image while a decoder reconstructs it using private key parameters. A PatchGAN discriminator and combined loss functions enhance security and training stability. Ding *et al.*^28^ proposed a deep learning-driven stream cipher (DeepKeyGen) that creates private keys by capturing image ‘styles’ within a transformation domain. A neural network maps a seed image into this domain to generate the key that is used to encrypt the medical image using the XOR operation. Wang and Zhang^29^ proposed an encryption strategy utilizing an Encryption Deep Neural Network (EDNN), where secret keys are generated using a Logistic map. The EDNN uses scrambled DCT coefficients as its weights, while a symmetric Decryption Deep Neural Network (DDNN) reconstructs the image by applying two pursuit algorithms aligned with the EDNN’s activation functions. Sang *et al.*^30^ introduced an encryption approach by utilizing the Logistic map and an autoencoder. First, the input image is scrambled by the Logistic map, then it is encoded by the autoencoder, producing the cipher image. Zhou *et al.*^31^ suggested a color image cryptosystem employing LSTM to train a hyperchaotic Lorenz system, generating pseudo-random signals. Combined with random numbers, these signals enhance security and key space, though the method targets smaller data volumes. Erkan *et al.*^32^ proposed an encryption strategy by combining CNN, log map, and bit reversion. The CNN extracts a public key from the input image to determine the initial conditions of the log map, which then generates a sequence used for encryption through a series of operations, including permutation, DNA encoding, diffusion, and bit reversal. Wang *et al.*^33^ suggested an encryption strategy for a medical image utilizing a V-net and a 4D hyperchaotic system. The V-net is trained to eliminate periodicity within the chaotic sequences, and the resulting sequences are used to diffuse image pixels for encryption. Ahmed *et al.*^2^ suggested an encryption scheme using DNA encoding, chaotic maps, and S-boxes. A CNN-based autoencoder is utilized to compress a 3D color image into a 2D grayscale image. This is followed by a DNA-based encryption that utilizes four chaotic maps, along with multiple S-boxes, to produce a ciphertext. Sun *et al.*^34^ presented an encryption strategy employing a lightweight VGG model to extract a key seed from the plain image, which is used to initialize a chaotic system. The encryption process begins by scrambling the image using a dynamic S-box, followed by further encryption with a single-connected (SC) layer and a VGG convolutional layer featuring a $1 \times 2$ kernel. The SC layer is dynamically made based on the secret key. Guo *et al.*^35^ introduced an encryption and compression framework using a reversed diffusion model. The outer diffusion encrypts the image while extracting semantic features, and the inner diffusion denoises and encodes it. A learning-based entropy coder compresses the encrypted image and features, enabling reconstruction via reverse encoding. Abed and Jawher^36^ presented a multi-chaos encryption scheme that integrates Block Compressive Sensing (BCS), Swin Transformer (ST), and Wild Horse Optimization (WHO). In this method, initial confusion is performed by DWT and FAN Transform. Then, it compresses $64\times 64$ image blocks using the Hadamard matrix and uses chaos numbers generated from ST for diffusion and WHO for scrambling. BCS is finally applied to boost speed and security. Huang *et al.*^37^ suggested an encryption framework, Chaos-Encoder Model, by combining CNNs and a transformer encoder. This model generates a unique data-dependent chaotic sequence. This sequence is then combined with a Lorenz hyperchaotic sequence to drive a multidirectional interleaved diffusion and a backtracking-based permutation process.

Table 1 presents a comparative analysis of the reviewed literature. Although numerous deep learning-based image encryption techniques have been proposed, most existing approaches suffer from limitations that hinder their broader applicability and efficiency. Many are designed for domain-specific scenarios, such as medical imaging^22,28,33^, and do not generalize well across diverse image types or application contexts. Furthermore, the complexity and computational overhead of architectures like GANs and transformers^21,26,36^ often restrict real-time deployment, especially on resource-constrained devices. While some methods introduce region-based or selective encryption^22,26^, there remains a lack of unified, adaptive frameworks that dynamically adjust encryption strength based on image content or user-defined sensitivity levels. Additionally, the integration of encryption with compression remains underexplored^35,36^, despite its importance for bandwidth-limited environments^38^. Most current methods also lack rigorous evaluation against modern threats such as adversarial attacks or deep learning-based reconstruction. These limitations highlight the need for a lightweight, content-aware image encryption framework that effectively balances security, adaptability, compression, and computational efficiency, while ensuring robustness against contemporary attack models and supporting cross-domain applications.

To address the limitations in existing deep learning-based image encryption methods, our work introduces several key innovations. A novel 6D hyperchaotic system is proposed to enhance security and unpredictability beyond traditional low-dimensional systems. The integration of ViTAE enables cross-domain generalization and efficient latent-space encryption, significantly reducing computational overhead and making the method suitable for resource-constrained environments. By focusing encryption on the latent representation, the approach also supports content-aware processing and potential downstream compression.

**Table 1:** Comparison of deep learning-based image encryption techniques.

Ref.	Model type	Chaotic component	Encryption strategy	Application / highlights
^2^	CNN Autoencoder	Multiple chaotic maps	DNA-based encryption + S-boxes	3D to 2D compression + encryption
^20^	Autoencoder + FFNN	Chaotic maps	Key generation via deep learning and chaos	Efficient key generation without iterative training
^21^	DCGAN	Hyperchaotic + Quaternion	Feistel network with quaternion key stream	High randomness and complexity
^22^	CycleGAN + ROI mining	–	Domain conversion + reconstruction	Medical image privacy
^23^	CNN	Chaotic maps	Dual-channel, adaptive diffusion + image fusion	Parallelism, optical/digital fusion
^24^	GAN	Logistic map	GAN-generated intermediate + XOR with chaos	Lightweight encryption
^25^	GAN	Chaotic system (1D)	Feistel network + DNA encoding	Block-wise encryption
^26^	GAN	Chaotic sequences	Selective scrambling + superposition diffusion	Efficient pixel-level diffusion
^27^	CycleGAN + Adversarial AE	–	Scrambling via encoder, reconstruct via decoder	Secure transmission with PatchGAN
^28^	DeepKeyGen	–	Style-based key generation + XOR	Medical image encryption
^29^	EDNN + DDNN	Logistic map	Scrambled DCT weights + symmetric decryption	Deep feature-based key generation
^30^	Autoencoder	Logistic map	Chaos-based scrambling + autoencoding	Lightweight encryption
^31^	LSTM	Lorenz hyperchaotic system	LSTM generates pseudo-random signals	Targeted at small data volumes
^32^	CNN	Log map	DNA encoding + permutation + bit reversal	Public key from image
^33^	V-Net	4D hyperchaotic system	Chaos-based pixel diffusion	Medical image encryption
^34^	Lightweight VGG	Chaotic system	S-box scrambling + SC layer + VGG convolution	Efficient mobile-friendly encryption
^35^	Reversed Diffusion Model	–	Outer diffusion (encryption) + inner diffusion (denoise) + compression	Joint encryption and compression
^36^	Swin Transformer + WHO + BCS	Multi-chaos + ST generated	DWT + FAN + WHO scrambling + BCS	High speed + block compression
^37^	CNN + Transformer Encoder	Lorenz hyperchaotic system	Chaos-Encoder Model (CEM) + multidirectional interleaved diffusion + backtracking-based permutation	Advanced multidirectional interleaved diffusion and permutation

## 6D hyperchaotic system

We present a novel 6D hyperchaotic system (6DHCS). This 6DHCS is represented mathematically by Eq. (1).

1 $$\begin{aligned} {\left\{ \begin{array}{ll} \dot{x_1}= -ax_2-ax_3-x_5-x_6\\ \dot{x_2}=bx_1-cx_2+x_5\\ \dot{x_3}= dx_1+x_1x_3+x_6 \\ \dot{x_4}= ex_1+x_2x_4-x_3\\ \dot{x_5}= fx_3+gx_5+x_2\\ \dot{x_6}=hx_5-ix_6\\ \end{array}\right. } \end{aligned}$$

where *a* to *i* denotes the system parameters and $x_i$, for $1 \le i \le 6$ are the state variables of the 6DHCS, and dot denotes derivatives with respect to time.

For a chaotic system of differential equations to be considered hyperchaotic, it must satisfy certain essential criteria^39^: (i) The system must have at least four dimensions, which our proposed system meets. (ii) It should contain at least two equations introducing instability, each incorporating at least one nonlinear term. As seen in Eq. (1), this requirement is satisfied by the equations for $\dot{x_3}$ and $\dot{x_4}$. (iii) The system must exhibit dissipativity, which is analyzed in the following subsection. Furthermore, the subsequent subsections analyze the suggested system’s dynamic behavior by assessing Lyapunov exponents (LEs), Kolmogorov entropy, the $0-1$ test, equilibrium points and stability, and the symmetry and invariance properties of the system. The NIST test also assesses the randomness of the sequences produced by the suggested 6DHCS system.

**Fig. 1:**
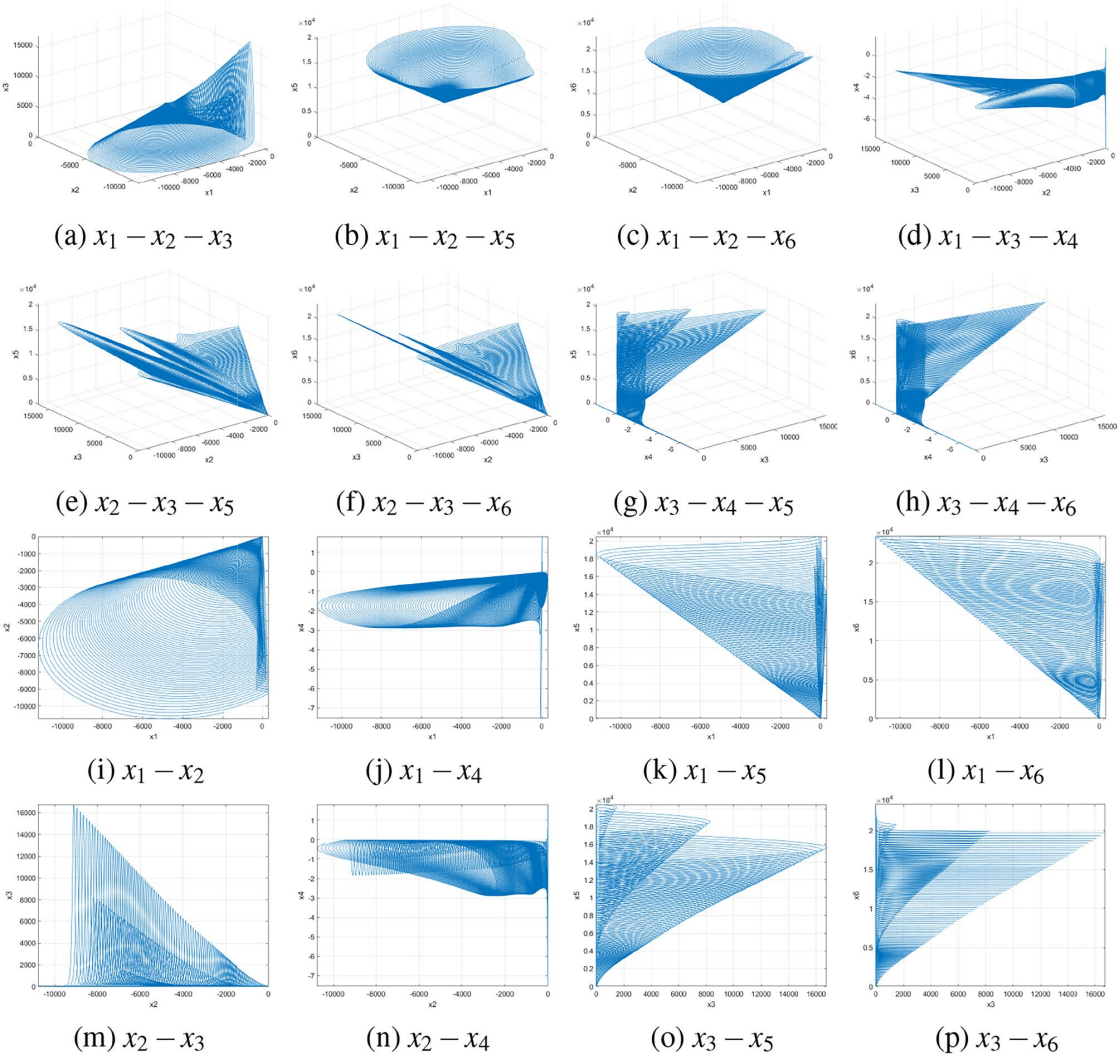
Attractors of the 6D hyperchaotic system.

### Dissipativity and attractor

Dissipativity and the presence of an attractor are important characteristics of a dynamic system, especially in the study of nonlinear chaotic behavior. Dissipativity refers to a system’s inclination to lose energy or contract toward a bounded region in phase space over time. A system is considered dissipative if the volume of its phase space decreases as time evolves, causing trajectories to converge within a limited portion of the state space.

An attractor represents a region in phase space to which the system’s trajectories eventually settle. These attractors can take the form of fixed points, periodic orbits (limit cycles), or chaotic (strange) attractors^40^.

To assess the dissipative property of the proposed 6DHCS, its divergence is computed, as shown in Eq. (2):

2 $$\begin{aligned} \displaystyle \Delta W=\frac{\partial \dot{x_1}}{\partial x_1}+\frac{\partial \dot{x_2}}{\partial x_2}+\frac{\partial \dot{x_3}}{\partial x_3}+\frac{\partial \dot{x_4}}{\partial x_4}+\frac{\partial \dot{x_5}}{\partial x_5}+\frac{\partial \dot{x_6}}{\partial x_6}=-(c-g+i) \end{aligned}$$

Using Liouville’s theorem^41^, the rate of change of the hypervolume *V*(*t*) of a smooth region $\Sigma (t)$ within $\mathbb {R}^6$ is expressed by Eq. (3):

3 $$\begin{aligned} \frac{dV(t)}{dt} = \int _{\Sigma (t)} ( - c + g - i ) \, dx_1 \, dx_2 \, dx_3 \, dx_4 \, dx_5 \, dx_6 = -(c - g + i)V(t) \end{aligned}$$

where $\Sigma (t) = \Sigma _0(t)$, with $\Sigma _0(t)$ representing the flow of *W*. When integrating with the initial volume *V*(0), the solution to Eq. (3) is:

4 $$\begin{aligned} V(t) = V(0) \exp \left( - (c - g + i) t \right) , \quad \text {for } t \ge 0. \end{aligned}$$

The system is classified as dissipative if the exponent in Eq. (4) is negative^41^. Since $c - g + i > 0$ (with $c = 0.08$, $g = 0.02$, and $i = 3.2$), the system meets the dissipativity condition. This indicates that the volume enclosing the system trajectories contracts exponentially as $t \rightarrow \infty$, with a decay rate of $-(c - g + i)$. As a result, the presence of an attractor in the proposed 6DHCS is assured.

### Lyapunov exponent

The rate of convergence or divergence of the neighboring trajectories of a dynamic system is measured by the Lyapunov exponent (LE). A system is stable if all LEs are negative, indicating that trajectories tend to settle toward equilibrium and are not sensitive to the initial conditions. In contrast, the presence of at least one positive LE, along with a negative total sum of all LEs, results in chaotic behavior characterized by exponential divergence and extreme sensitivity to the initial conditions. When two or more LEs are positive, the system is classified as hyperchaotic, indicating greater complexity and higher-dimensional instability. The LEs of the proposed 6DHCS with parameters set as $a = 6.5,\, b = 3.5,\, c = 0.08,\, d = 6,\, e = 2,\, f = 22,\, g = 0.02,\, h = 3.9,\, i = 3.2$ are: $LE_1 = 1.891695,\quad LE_2 = 1.890940,\quad LE_3 = 0.001818,\quad LE_4 = -0.494647,\quad LE_5 = -3.274836,\quad LE_6 = -3.274970.$ The analysis reveals three positive and three negative LEs, with a total sum of $L = -3.26$. Since $L < 0$, the system exhibits hyperchaotic behavior. The attractor of the 6DHCS, shown in Fig. 1, is generated using the specified parameter values and initial conditions $x_i(0)=1$, for $1\le i\le 6$. The figure confirms the system’s dynamics across various trajectories.

The Kaplan-Yorke dimension, usually referred to as the Lyapunov dimension, estimates the complexity or fractal nature of an attractor in a dynamic system. It is defined by Eq. (5):

5 $$\begin{aligned} D_{KY} = l + \frac{1}{|LE_{l+1}|} \sum _{j=1}^{l} LE_j \quad \text {such that} \quad \sum _{j=1}^{l} LE_j > 0 \quad \text {and} \quad \sum _{j=1}^{l+1} LE_j < 0 \end{aligned}$$

**Fig. 2:**
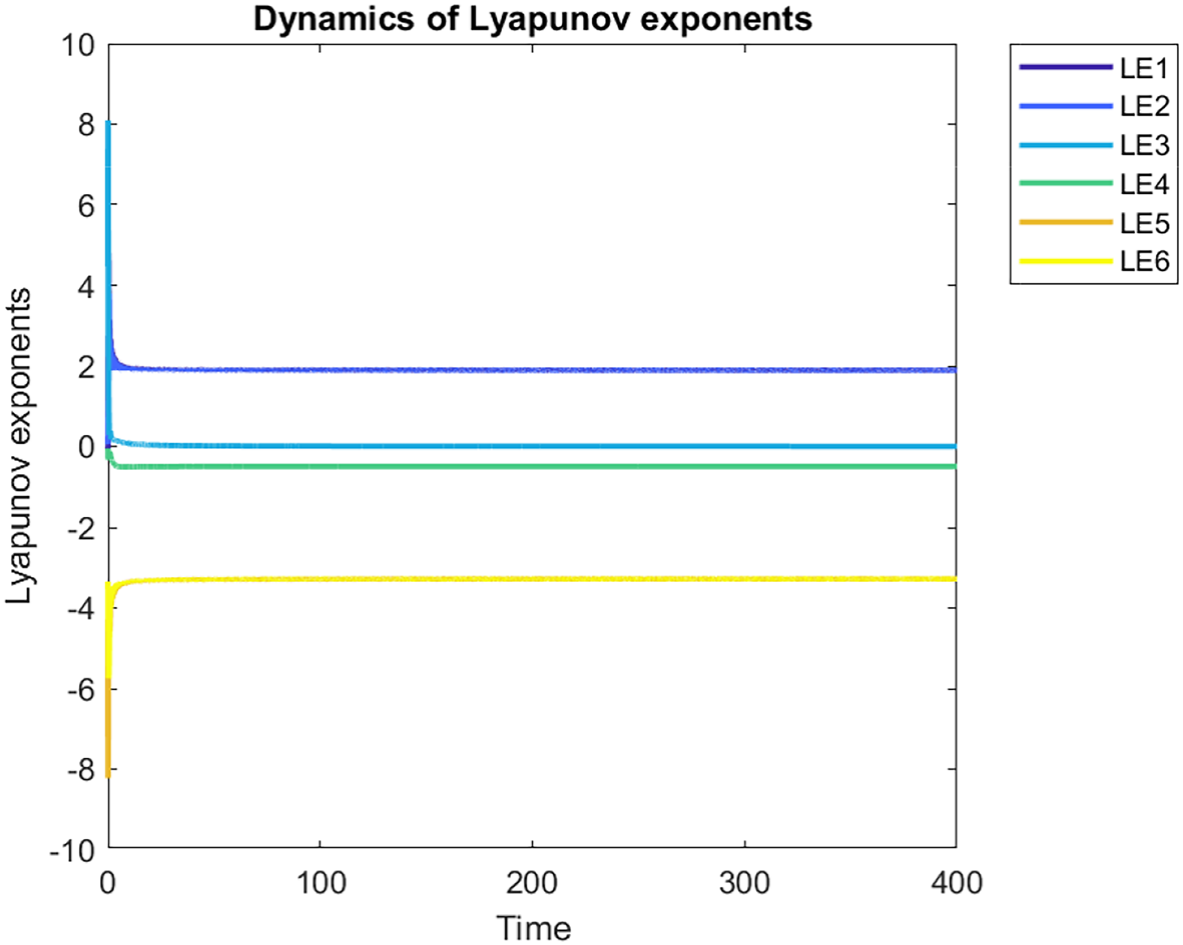
Lyapunov exponents of the 6DHCS at time $\in [0,400]$, step$=0.02$.

Here, $l=5$ and $D_{KY}=5+\frac{LE_1+LE_2+LE_3+LE_4+LE_5}{|LE_6|}=5.0046$. The fractional value of $D_{KY}$ indicates the presence of a strange attractor, characterizing the system as highly complex and chaotic with fractal-like geometry^42^. Fig. 2 illustrates the Lyapunov exponents of the proposed 6DHCS.

### Kolmogorov entropy

Kolmogorov entropy (KE), also known as metric entropy, is a quantitative measure of the complexity and unpredictability of a dynamic system. It describes how quickly a system generates new information or becomes unpredictable over time^43^. The KE can be estimated using the positive Lyapunov exponents through Pesin’s theorem, expressed by Eq. (6):

6 $$\begin{aligned} H_{KE}=\sum _{LE>0} LE \end{aligned}$$

where *LE* refers to the Lyapunov exponents. This formulation establishes a relation between the dynamic instability of the system and the rate of information generation. A higher KE value implies stronger chaotic behavior, as minor changes in initial conditions result in rapid divergence of trajectories, increasing system unpredictability.

In this work, the KE is calculated to assess the chaotic intensity of the proposed 6DHCS based on the obtained LEs. Considering only the positive LEs, the $KE = LE_1+LE_2+LE_3 = 3.7844$. The relatively high KE value demonstrates that the proposed 6DHCS exhibits strong chaotic dynamics, high sensitivity to initial conditions, and enhanced randomness. Furthermore, we compared the KE values with the existing chaotic maps in the literature, which are reported in Table 2. From the table, it is shown that the proposed 6DHCS achieves a noticeably higher entropy, confirming its superior capability to generate more complex and unpredictable behavior. This enhanced chaotic property makes it highly effective for secure cryptographic and image encryption applications.

**Table 2:** Kolmogorov entropy comparison.

Chaotic System	LEs				KE
In^21^	$LE_1 = 2.8747$,	$LE_2 = 0.0523$,	$LE_3 = -0.0078$,	$LE_4 = -23.9192$	2.9270
In^31^	$LE_1 = 0.3381$,	$LE_2 = 0.1586$,	$LE_3 = 0$,	$LE_4 =-15.1752$	0.4967
In^33^	$LE_1= 0.343$,	$LE_2= 0.052$,	$LE_3 = -0.305$,	$LE_4 = -36.640$	0.3950
In^34^	$LE_1=0.4222$,	$LE_2 = 0.0698$,	$LE_3 = -4.0025$,	$LE_4 = -5.3752$	0.4920
Proposed	$LE_1 = 1.8917$,	$LE_2 = 1.8909$,	$LE_3 = 0.0018$,	$LE_4 = -0.4946$,	3.7844
	$LE_5 = -3.2748$,	$LE_6 = -3.2749$			

### 0-1 test

The 0-1 test is used further to analyze the chaotic behavior of the proposed 6DHCS. The 0-1 test is a mathematical tool for distinguishing between regular and chaotic dynamics directly from a scalar time series, without phase-space reconstruction. This test analyzes the growth behavior of a transformed trajectory derived from the observed data, rather than calculating Lyapunov exponents.

For a time series $\phi (i), i=1, 2,..., N$, the test defines a pair of translation variables ((*p*(*n*), *q*(*n*))) as follows:

$$\begin{aligned} p(n)=\sum _{i=1}^{n}\phi (i)\cos (ic), \quad q(n)=\sum _{i=1}^{n}\phi (i)\sin (ic) \end{aligned}$$

where $c\in (0,\pi )$ is a randomly picked constant. The trajectory in the $p-q$ plane remains bounded if the underlying dynamics are regular. In contrast, chaotic dynamics lead to Brownian-like, unbounded diffusion^44^.

To classify this behaviour quantitatively, the mean square displacement $(M_{c}(n))$ is computed:

$$\begin{aligned} M_c(n) = \frac{1}{N-n} \sum _{i=1}^{N-n} \left[ (p(i+n)-p(i))^2+(q(i+n)-q(i))^2\right] \end{aligned}$$

For regular motion, $M_{c}(n)$ stays bounded, while for chaotic dynamics it scales linearly with *n*. The asymptotic growth rate is estimated via the correlation-based coefficient $K_c$. To eliminate pathological selections of *c*, we repeat the test with $N_c$ random selections of *c* (here $N_c = 300$) and then get the final result by:

$$\begin{aligned} K = \text {median}\{K_c\}\end{aligned}$$

A value of $K \approx 0$ indicates non-chaotic behaviour, whereas $K \approx 1$ confirms chaos^45^.

**Fig. 3:**
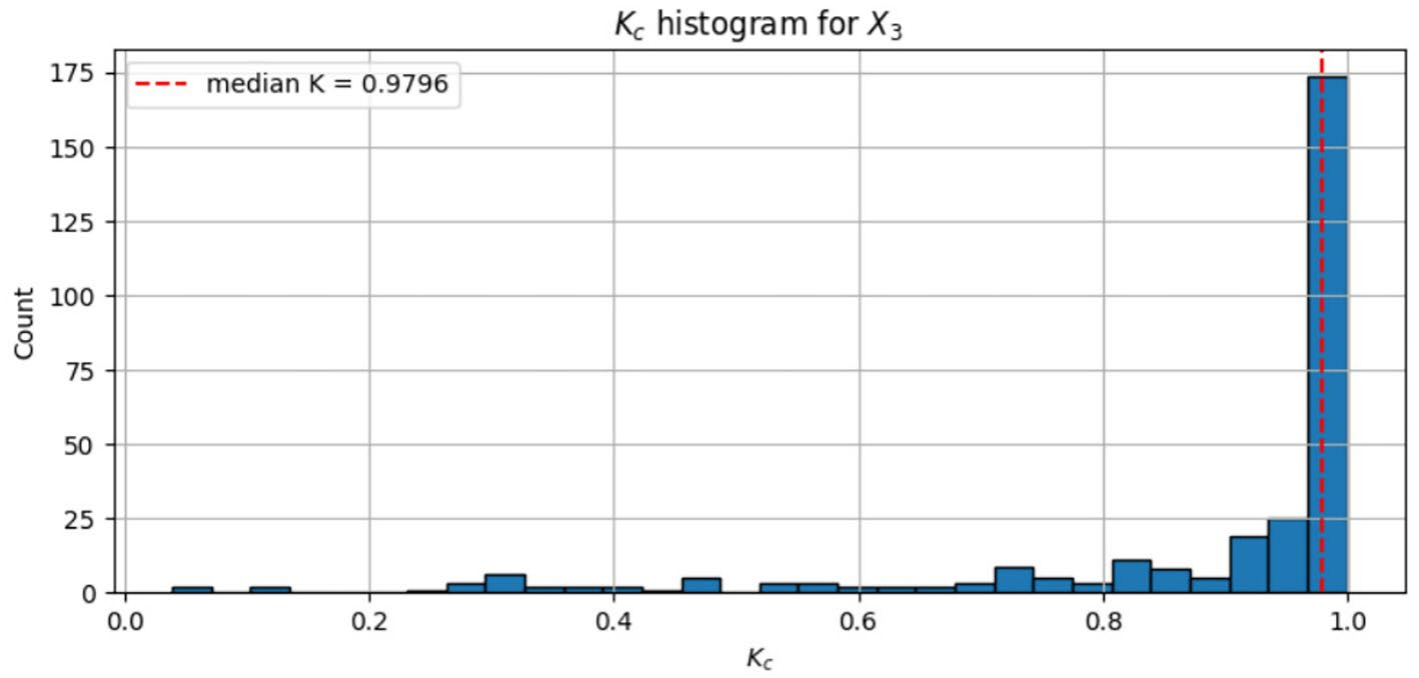
Histogram of $K_c$ for $X_3$.

**Fig. 4:**
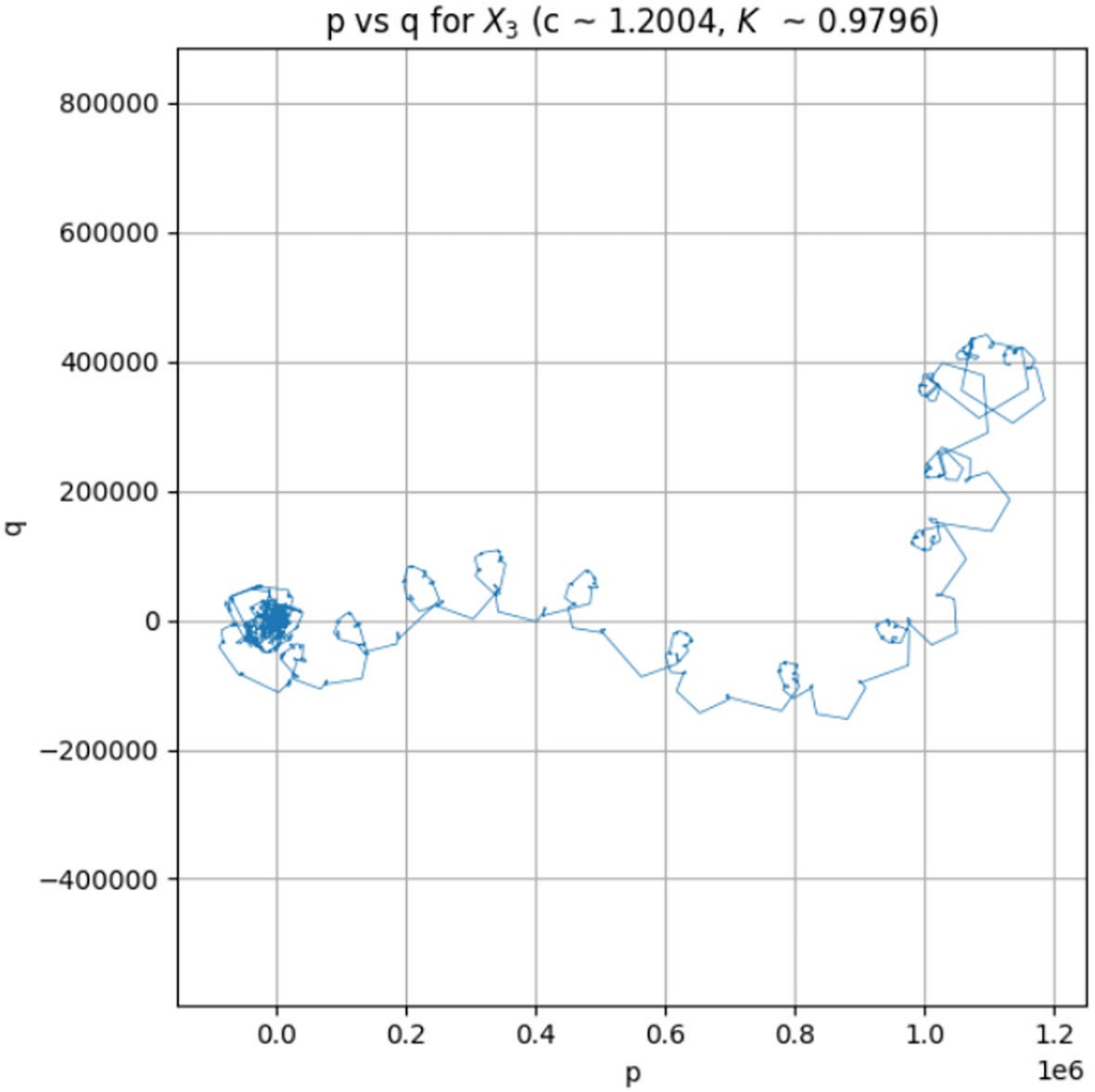
Diffusive trajectory in the $p-q$ plane for $X_3$ corresponding to $K = 0.9796$.

In the current research, the 0-1 test is conducted on the $X_3$ sequence of the 6DHCS, using 13,000 post-transient data values. The median correlation coefficient is $K = 0.9796$, indicating strong chaos. The histogram of $K_c$ values is also very concentrated at unity as shown in Fig. 3, indicating the presence of hyperchaotic dynamics. Also, the curve in the $p-q$ plane exhibits a diffusion-like structure of chaotic motion as illustrated in Fig. 4. Thus, the findings of the 0-1 test are entirely consistent with the multi-positive Lyapunov exponents and confirm that the complex, high-dimensional hyperchaos characterizes the suggested system.

### Equilibrium point and stability

Equilibrium points (EPs) are essential for analysing the dynamics of nonlinear systems, as they offer valuable information about the system’s stability. They are determined by equating the system’s differential equations to zero^46^.

For this system, two equilibrium points are identified: $EP_0=[0,0,0,0,0,0]$ and $EP_1=$ $[35.5797, 43.1810, -1.8527, -1.6908$, $-121.0744, -147.5595]$. The Jacobian matrix (J) for the system (1) is given by:

$$\begin{aligned} J= \begin{bmatrix} 0& -a& -a& 0& -1& -1\\ b& -c& 0& 0& 1& 0\\ d+x_3& 0& x_1& 0& 0& 1\\ e& x_4& -1& x_2& 0& 0\\ 0& 1& f& 0& g& 0\\ 0& 0& 0& 0& h& -i \end{bmatrix} = \begin{bmatrix} 0& -6.5& -6.5& 0& -1& -1\\ 3.5& -0.08& 0& 0& 1& 0\\ 6+x_3& 0& x_1& 0& 0& 1\\ 2& x_4& -1& x_2& 0& 0\\ 0& 1& 22& 0& 0.02& 0\\ 0& 0& 0& 0& 3.9& -3.2 \end{bmatrix} \end{aligned}$$

The Jacobian matrices of the system (1) evaluated at the equilibrium points $EP_0$ and $EP_1$ are expressed as follows:

$$\begin{aligned} J_{EP_0}= \begin{bmatrix} 0& -6.5& -6.5& 0& -1& -1\\ 3.5& -0.08& 0& 0& 1& 0\\ 6& 0& 0& 0& 0& 1\\ 2& 0& -1& 0& 0& 0\\ 0& 1& 22& 0& 0.02& 0\\ 0& 0& 0& 0& 3.9& -3.2 \end{bmatrix} ~~~~~~~~~~~~~~~~~J_{EP_1}= \begin{bmatrix} 0& -6.5& -6.5& 0& -1& -1\\ 3.5& -0.08 & 0 & 0 & 1 & 0\\ 4.1473& 0& 35.5797& 0& 0 & 1\\ 2& -1.6908& -1& 43.1810& 0& 0\\ 0& 1& 22& 0& 0.02& 0\\ 0& 0& 0& 0& 3.9& -3.2 \end{bmatrix} \end{aligned}$$

The eigenvalues at the equilibrium points $EP_0$ and $EP_1$ are presented in Table 3. It is shown that, at the equilibrium point $EP_0$, the system exhibits two eigenvalues, $\lambda _1$ and $\lambda _2$, with positive real parts. Similarly, at $EP_1$, the system has two positive eigenvalues, $\lambda _1$ and $\lambda _2$. Since the positive eigenvalue is present for all equilibrium points, each point is an unstable saddle-focus point^41^.

**Table 3:** Eigenvalues at equilibrium points $EP_0$ and $EP_1$.

$EP_0$				$EP_1$			
$\lambda _{1,2}=$ $1.89006 \pm 6.94724 i$		$\lambda _5=$ $-0.494367$		$\lambda _1=$ 43.181		$\lambda _{3,4}=$ $-0.086546 \pm 4.12118 i$	
$\lambda _{3,4}=$ $-3.27288\pm 4.66569 i$		$\lambda _6=$ 0		$\lambda _2=$ 34.7886		$\lambda _{5,6}=$ $-1.1479 \pm 0.300725 i$	

### Symmetry and invariance

The 6DHCS exhibits invariance under the coordinate transformation

$$\begin{aligned} (x_1, x_2, x_3, x_4, x_5, x_6) \rightarrow (-x_1, -x_2, -x_3, x_4, -x_5, -x_6) \end{aligned}$$

This transformation indicates that the system possesses symmetry with respect to the $x_4$-axis. As a result, for every significant trajectory, there exists a corresponding symmetric trajectory, reflecting the inherent symmetric nature of the proposed system.

### Randomness test for chaotic sequences

To assess the randomness, the SP800-22 test suite proposed by NIST is employed^47^. This suite includes 15 primary tests along with 188 supplementary ones. A sequence is considered to pass a given test if the corresponding P-value exceeds 0.01; otherwise, the sequence is regarded as potentially predictable. P-values serve as a critical statistic for measuring the performance of time series. If every test succeeds, the sequence randomness criteria are met. For the evaluation, six distinct sequences, each comprising $10^8$ points, are generated using the 6DHCS. The initial parameters are set to $a=6.5,\ b=3.5,\ c=0.08,\ d=6,\ e=2,\ f=22,\ g=0.02,\ h=3.9,\ i=3.2$, for $1 \le i \le 6$ and simulation time spanning the interval [0, 400]. Table 4 presents the results of the NIST test suite, including the respective P-values. The results confirm that all generated sequences successfully passed the tests, demonstrating strong pseudo-random characteristics.

**Table 4:** NIST randomness test of the 6D hyperchaotic system.

Statistical test	$X_1$	$X_2$	$X_3$	$X_4$	$X_5$	$X_6$	Remark
Frequency (Monobit)	0.7535	0.0424	0.9952	0.3919	0.2855	0.0851	$\checkmark$
Frequency Test within a Block	0.2319	0.2855	0.1744	0.9418	0.7998	0.9348	$\checkmark$
Runs	0.1651	0.4167	0.3544	0.4480	0.9465	0.6679	$\checkmark$
Longest-Run-of-Ones in a Block	0.1424	0.9137	0.5062	0.0695	0.0266	0.2841	$\checkmark$
Binary Matrix Rank	0.8796	0.66181	0.2943	0.2132	0.4963	0.0549	$\checkmark$
Spectral	0.8890	0.3568	0.2249	0.3953	0.9560	0.9560	$\checkmark$
Non-overlapping Template Matching	0.6825	0.5067	0.7783	0.8078	0.3494	0.5334	$\checkmark$
Overlapping Template Matching	0.9856	0.8357	0.1745	0.3964	0.8149	0.5544	$\checkmark$
Maurer’s “Universal Statistical”	0.7504	0.2339	0.0631	0.3737	0.1681	0.0287	$\checkmark$
Linear Complexity	0.4173	0.1617	0.2319	0.5114	0.1563	0.1364	$\checkmark$
Statistical	0.3633	0.0919	0.6525	0.5209	0.5642	0.2075	$\checkmark$
	0.1651	0.4191	0.3554	0.4496	0.9489	0.6715	$\checkmark$
Approximate Entropy	0.6642	0.1093	0.8852	0.5689	0.5097	0.3584	$\checkmark$
Cumulative Sums (Cusums)	0.6374	0.0291	0.9532	0.5407	0.4791	0.1269	$\checkmark$
	0.6918	0.5306	0.9781	0.7872	0.6976	0.5463	$\checkmark$
Random Excursions	0.0735	0.1226	0.1153	0.2399	0.2041	0.3312	$\checkmark$
Random Excursions Variant	0.0113	0.1744	0.3580	0.1790	0.0995	0.0352	$\checkmark$

## Vision transformer autoencoder

**Fig. 5:**
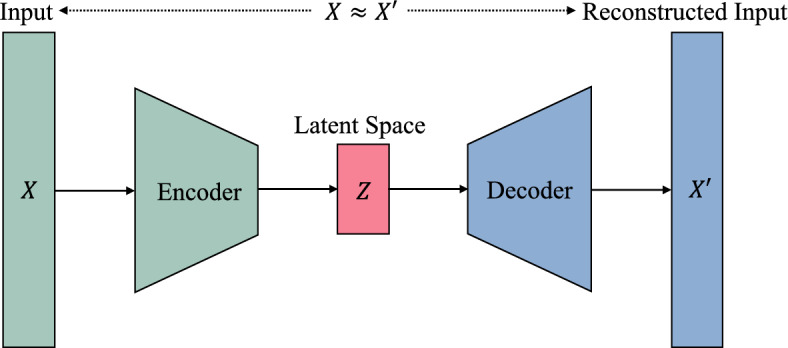
Basic structure of autoencoder.

This section presents the Vision Transformer Autoencoder (ViTAE), a Transformer-based architecture tailored for high-fidelity image reconstruction. In contrast to standard convolutional or recurrent autoencoders, ViTAE substitutes the normal encoding and decoding processes with Transformer blocks that utilize multi-head self-attention (MSA). This technique allows the model to capture both fine-grained features and global contextual information within images, thereby improving its ability to generalize and reconstruct effectively. The model adheres to a conventional autoencoder architecture, comprising an encoder that transforms the input into a compact latent space and a decoder that reconstructs the image from this representation. Fig. 5 illustrates the basic framework of an autoencoder, where *X* denotes the original input, *Z* represents the latent space, and $X'$ indicates the reconstructed input.

**Fig. 6:**
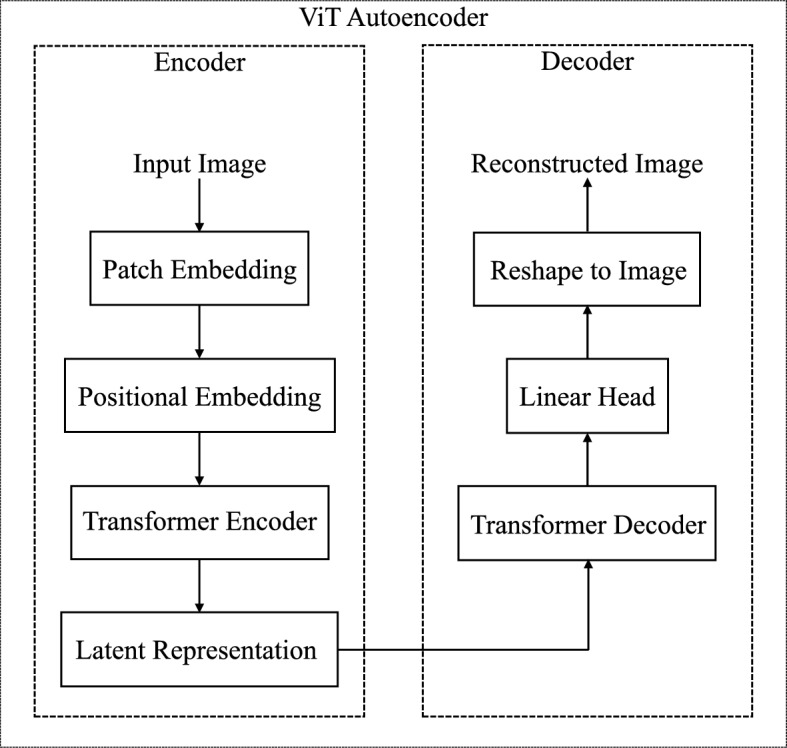
Architectural design of the ViT Autoencoder.

### Model architecture

The ViTAE architecture strictly follows the autoencoder paradigm, in which the input image is first transformed into a compact latent representation and then reconstructed into a high-resolution output through the decoding process. However, unlike traditional CNN-based autoencoders, ViTAE is entirely built using Transformer components. This section presents an in-depth explanation of each component of the architecture, covering the process from image tokenization to complete reconstruction. Fig. 6 illustrates the comprehensive architectural layout of the ViT-based autoencoder.

#### Encoder

The encoder processes input images through the following sequential operations:

- Input Image: The model takes a color image $X \in \mathbb {R}^{P \times Q \times 3}$ as input. Here, we explain the method with $P=Q=256$, that is, the image is of size $256 \times 256 \times 3$.
- Patch Embedding: During the patch embedding phase, the input image is segmented into non-overlapping patches of size $32 \times 32$, yielding a total of 64 patches per image. Each patch retains the three RGB channels; the flattened representation of each patch comprises $32 \times 32 \times 3 = 3072$ pixel values. After that, these flattened vectors are passed through a linear projection layer that maps the 3072-dimensional inputs into an embedding space of size 2048. This projection is achieved through a convolutional layer, where both the kernel size and stride are set to 32, matching the patch dimensions, ensuring spatial non-overlap and preserving local patch structure. Consequently, the 2D image is converted into a 1D sequence of 64 embedded tokens, each having a dimensionality of 2048, making it compatible for input into the Transformer encoder.
- Positional Embedding: Since Transformers are inherently permutation-invariant and lack built-in positional awareness, it is necessary to explicitly inject positional information to retain the spatial structure of the input image. To address this, ViTAE incorporates learnable positional embeddings with the patch embeddings before passing them to the encoder. The resulting combination of patch and positional embeddings constitutes the input sequence for the Transformer encoder.
- Transformer Encoder: The ViTAE encoder module comprises six stacked encoder layers. Each layer includes a multi-head self-attention (MSA) mechanism with four attention heads, followed by a multi-layer perceptron (MLP) block with an expansion factor of two, resulting in a hidden dimension of 4096. Every component is accompanied by layer norm and residual connections to enable effective and stable training. The encoder simultaneously processes the complete sequence of 64 patches, allowing the model to learn both local features and global dependencies across the entire image. This design enables the encoder to produce a compact and informative latent representation, preserving essential semantic and spatial features required for accurate reconstruction.
- Latent Representation: The encoder component produces a latent representation of the input image ($256 \times 256 \times 3$), comprising 64 tokens, each with a dimensionality of 2048. Therefore, the dimension of the latent image is $64 \times 2048$. This latent space encapsulates the most salient visual and spatial characteristics of the input while significantly diminishing its dimensional complexity. It serves as a bottleneck in the autoencoder system and is provided as input to the decoder for image reconstruction.

#### Decoder

The decoder reconstructs images from the latent representation through the following operations:

- Transformer Decoder: The decoder module in ViTAE comprises six stacked transformer decoder layers, each designed to reconstruct the image from the latent representation produced by the encoder. Unlike standard auto-regressive decoders, no masking is applied in the self-attention mechanism^17,18^, as the entire sequence is processed in parallel for image reconstruction. Each decoder layer starts with a multi-head self-attention mechanism comprising four heads, allowing tokens to interact across the entire sequence. This is followed by layer normalization to promote stable training. Subsequently, a multi-head cross-attention mechanism, also with four heads, is applied, allowing the decoder to attend to the encoder’s output (treated as memory), thereby incorporating global contextual information from the input image. An additional layer of normalization is used next to prepare the token representations for the feed-forward network (FFN), which expands the intermediate dimensionality to 4096, enabling the model to capture subtle variations within each token better. The decoder block is further enhanced with an additional layer of normalization to ensure uniformity in the output. Residual connections are incorporated around each sub-module in the block to facilitate the efficient training of deeper layers without degradation and to help maintain a steady gradient flow. This structure facilitates the decoder to generate rich, spatially coherent features for high-fidelity image reconstruction.
- Linear Head: Following the final decoder layer, each of the 64 output tokens, each with 2048 dimensions, is passed through a linear projection layer. This head maps each token back into a 3072-dimensional vector, corresponding to a flattened $32 \times 32 \times 3$ patch, thereby reconstructing the original pixel values of the input patch.
- Reshape to Image: The 64 reconstructed patch vectors are reshaped into their original 2D spatial form and reassembled into the full image layout. This step restores the image to its original structure, maintaining the spatial relationships between patches.
- Reconstructed Image: The final result is a reconstructed image of the shape $256 \times 256 \times 3$. This image aims to closely match the original input in both appearance and structure, completing the autoencoding process. However, although it looks similar to the original image, it is not an exact replica of it,as the ViTAE architecture is inherently lossy.

### Loss function and training strategy

To assess the accuracy of the rebuilt images, the MSE loss function is employed. This function measures the pixel-wise difference between the input and rebuilt images, thus serving as an indicator of reconstruction fidelity. The MSE loss function is given by

$$\begin{aligned} \text {MSE} = \frac{1}{N} \sum _{i=1}^{N} (X_i - \hat{X}_i)^2 \end{aligned}$$

where *N* denotes the total number of pixels, $X_i$ and $\hat{X}_i$ denote the pixel values of the input and reconstructed images, respectively. The model reduces the reconstruction error and improves the perceptual similarity between the output and the ground-truth images by minimizing this loss.

The ViTAE model is implemented in the Kaggle environment using PyTorch and trained with the AdamW optimizer, configured with a learning rate of $1\times 10^{-5}$ and a weight decay of 0.01. To enhance computational efficiency and reduce memory consumption, mixed precision training is applied using GradScaler, which dynamically scales the loss to prevent underflow during backpropagation. The model is trained for 100 epochs with a batch size of 4, leveraging two T4 GPUs through parallel to distribute the workload across both GPUs. This parallelization significantly accelerated the training process while ensuring consistent performance and stability. The two NVIDIA T4 GPUs, each with 15GB of memory, enabled efficient execution of large-scale computations. The training phase exhibited smooth convergence, with the reconstruction loss progressively decreasing over successive epochs, indicating the model’s effective learning and improved reconstruction accuracy.

### Dataset

We used the Animal Faces HQ (AFHQ) dataset^48^, which comprises 15,000 high-quality images with a resolution of $512 \times 512 \times 3$ pixels, to train and evaluate the proposed ViTAE model. The dataset comprises a diverse range of facial features, fur textures, colors, and backdrop contexts in three visually distinct domains: cats, dogs, and wildlife. This rich visual diversity makes AFHQ a particularly suitable benchmark for evaluating the effectiveness of image reconstruction models. The dataset is partitioned into a training set and a testing set to enable thorough and domain-balanced evaluation. A total of 13,500 images are used for training, with 4,500 images from each of the three categories contributing to the training set. For testing purposes, the final 500 images per domain, totaling 1,500, are set aside. In addition, the heterogeneity inherent in the dataset encourages the model to learn discriminative features that generalize well beyond a narrow set of visual patterns, thus supporting a comprehensive assessment of reconstruction quality under varied real-world conditions.

### Preprocessing and augmentation

Several preprocessing steps are performed before training the model in order to standardize and optimize the input images for efficient training. All images are resized to a consistent resolution of $256 \times 256 \times 3$ pixels, ensuring homogeneity throughout the dataset and reducing memory overhead during training. Due to resource constraints, training the color images of size $512 \times 512 \times 3$ on platforms like Kaggle is not feasible. Furthermore, pixel values are normalized to the range $[-1, 1]$ with a zero mean and 0.5 standard deviation for each channel, which is a typical formalization method in deep learning pipelines to stabilize training dynamics.

Several data augmentation techniques are used during training to increase the model’s resilience and generalization capacity. To add spatial diversity and aid the model in learning features that are invariant in left-right orientation, each image had a 50% chance of being flipped horizontally. In addition, random rotations of up to 15 degrees are applied to account for variations in viewpoint and alignment. To simulate positional displacements, the images are also randomly translated in both vertical and horizontal directions by up to 10%. Together, these augmentation techniques improve the variations in the training dataset, thwarting the overfitting issue, and allowing the model to reconstruct the images more precisely.

### Performance evaluation of ViTAE

The ViTAE model demonstrated effective convergence, consistently reducing the reconstruction error throughout the training process. The ViTAE model achieved an average MSE loss of approximately 0.0021 over 100 epochs, demonstrating a high reconstruction accuracy.

To assess the perceptual quality of the reconstructed images, several metrics are computed, including the Peak Signal-to-Noise Ratio (PSNR), Structural Similarity Index (SSIM)^49^, Multi-Scale Structural Similarity Index (MS-SSIM)^50^, Feature Similarity Index (FSIM)^51^, and Visual Information Fidelity (VIF)^52^. The results for six test images (‘Cat’, ‘Dog’, ‘Cheetah’, ‘Lion’, ‘Tiger’, and ‘Wolf’) are summarized in Table 5. As observed, the ViTAE model effectively reconstructed the images, achieving an average PSNR of 33.3447, SSIM of 0.9334, MS-SSIM of 0.9805, FSIM of 0.9998, and VIFP of 0.8484, indicating excellent perceptual fidelity and structural preservation.

A compact latent representation of size $64 \times 2048$ is constructed by encoding the original color image of size $256 \times 256 \times 3$. The autoencoder model achieves a compression ratio $\frac{256\times 256 \times 3}{64 \times 2048}$ = $\frac{3}{2}$ = 1.5:1, that is, $\frac{1}{3}$ the data from the original image can be reduced. The proposed compression achieves bit-per-pixel (bpp) = $\frac{64 \times 2048}{256 \times 256}$ = 16; in the original image bpp is 24 bits. 16 bpp is not as compact as extreme-low-bitrate compression techniques, but it has numerous significant advantages. It maintains an optimal balance between compression and visual quality while preserving fine-grained structural and perceptual details. This level of compression is beneficial for applications that require high reconstruction fidelity, such as biometric systems, satellite image processing, and specific medical imaging, where an exact pixel-to-pixel match is not necessary.

According to our proposed VitAE model, the compression ratio remains fixed at 1.5:1. We can increase this ratio by increasing the block size (i.e., decreasing the number of encoded blocks) or reducing the dimensionality of the embedding space (e.g., reducing the 2048-dimensional latent vector), thus enabling a more compact latent representation. These alterations compromise reconstruction quality; as the compression ratio increases, it loses fine-grained structural and perceptual details. Consequently, a trade-off arises between compression efficiency and output fidelity, which must be carefully balanced depending on the requirements of the target application.

To further validate the performance of the ViTAE model, a comparative analysis is performed with a CNN-based autoencoder trained on the same AFHQ dataset. The CNN model consists of a symmetric encoder–decoder architecture with multiple convolutional and transposed convolutional layers. The encoder part consists of six convolutional layers with progressively increasing filter sizes (from 64 to 2048) to extract hierarchical image features while reducing spatial resolution from $256 \times 256$ to $8 \times 8$. The latent representation is thus formed with a dimension of $2048 \times 8 \times 8$, flattened into a vector of size $2048 \times 64$. The decoder replicates the structure of the encoder, employing transposed convolutional layers to reconstruct the original image from its compressed latent features progressively. For stable and efficient learning, ReLU activations and batch normalization are used. The AdamW optimizer with a learning rate of $1\times 10^{-5}$ and a weight decay of 0.01, is used to optimize the model. Training is conducted for 100 epochs with a batch size of 4, using the Mean Squared Error (MSE) loss function to effectively reduce reconstruction errors between the input and output images.

The CNN-based autoencoder achieved an average PSNR of 31.0460, SSIM of 0.8921, MS-SSIM of 0.9731, FSIM of 0.9990, and VIFP of 0.6923. These values are lower across all metrics compared to the VitAE model, highlighting that ViTAE provides superior reconstruction quality and perceptual consistency.

**Fig. 7:**
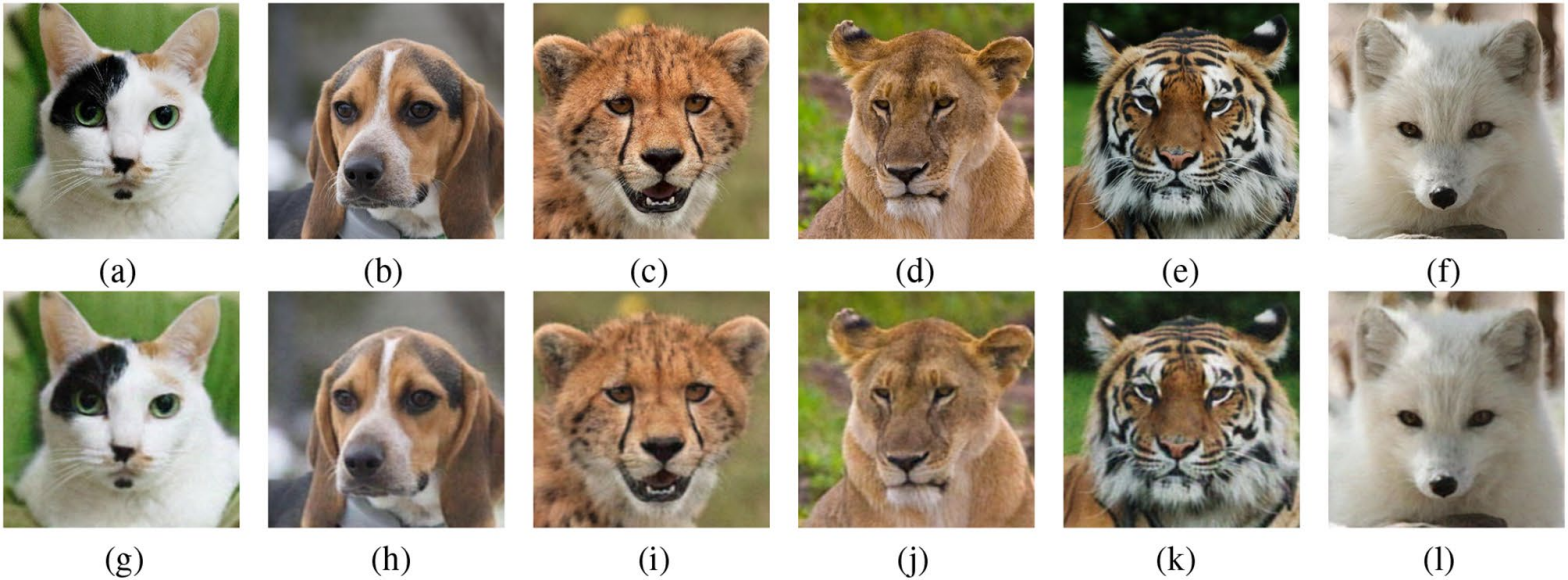
Output of the Vision Transformer Autoencoder: (**a**) - (**f**) Original images, (**g**) - (**l**) reconstructed images.

The reconstructed output of the original images by ViTAE is illustrated in Fig. 7, showcasing its capability to generate visually accurate and high-quality images. The ViTAE model offers several advantages over traditional convolutional autoencoders, including global context modeling through self-attention mechanisms, which enables the network to capture long-range dependencies more effectively. Additionally, the use of multi-head attention improves feature representation, leading to more precise and coherent reconstructions. The superior reconstruction accuracy and robustness of the model make it a promising solution for high-fidelity image restoration tasks, with potential applications in medical imaging, satellite image processing, and other fields where detailed image reconstruction is required but an exact match is not mandatory.

**Table 5:** Perceptual quality evaluation of reconstructed images using PSNR, SSIM, MS-SSIM, FSIM and VIFP metrics.

Image	VITAE					CNN-based autoencoder				
	PSNR	SSIM	MS-SSIM	FSIM	VIFP	PSNR	SSIM	MS-SSIM	FSIM	VIFP
Cat	33.4542	0.9226	0.9774	0.9998	0.8342	31.8962	0.8950	0.9767	0.9994	0.7073
Dog	33.8042	0.9217	0.9801	0.9998	0.8239	30.5476	0.8825	0.9727	0.9991	0.6827
Cheetah	32.5812	0.9397	0.9828	0.9996	0.8128	30.4012	0.9127	0.9755	0.9987	0.6673
Lion	32.3492	0.9264	0.9769	0.9997	0.8126	29.9141	0.8391	0.9620	0.9983	0.6283
Tiger	32.2845	0.9451	0.9804	0.9997	0.8637	30.6345	0.9009	0.9756	0.9991	0.7313
Wolf	35.5950	0.9448	0.9852	0.9999	0.9433	32.8826	0.9222	0.9763	0.9995	0.7367
Average	33.3447	0.9334	0.9805	0.9998	0.8484	31.0460	0.8921	0.9731	0.9990	0.6923

## Proposed image encryption method

The study introduces a novel encryption algorithm that integrates a 6D hyperchaotic system (6DHCS) with ViTAE. Initially, this method compresses the plain image through an autoencoder. The resulting compressed image is then encrypted using the hyperchaotic system. The overall process, which involves encoding the image followed by encryption, takes less time. Before detailing the encryption and decryption procedures, the following subsection discusses two contributions to achieve confusion and diffusion in the proposed method: the Random Shuffle technique and the Trifid Cipher transformation.

### Random shuffle technique

The Fisher-Yates shuffle (FYS), also called the Knuth Shuffle algorithm^53,54^, is a classical and widely used method to produce a random permutation of a finite sequence. The algorithm operates by iterating backward through the array, starting from the last element down to the first. The current element of the array is swapped with another element, which is located in any position from the first position to the current position. For each step, it ensures a uniform distribution across all possible permutations and makes every permutation equally likely. This method is valuable for its computational efficiency, reaching a linear time complexity of *O*(*n*), where *n* is the total number of elements. With its simple logic and great statistical dependability, the algorithm is well-suited for a wide range of tasks, from simulations and randomized algorithms to neural network tasks, such as shuffling data. The Fisher-Yates shuffle is described in the following pseudocode:
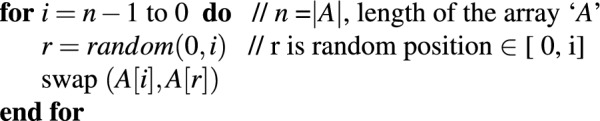

**Fig. 8:**
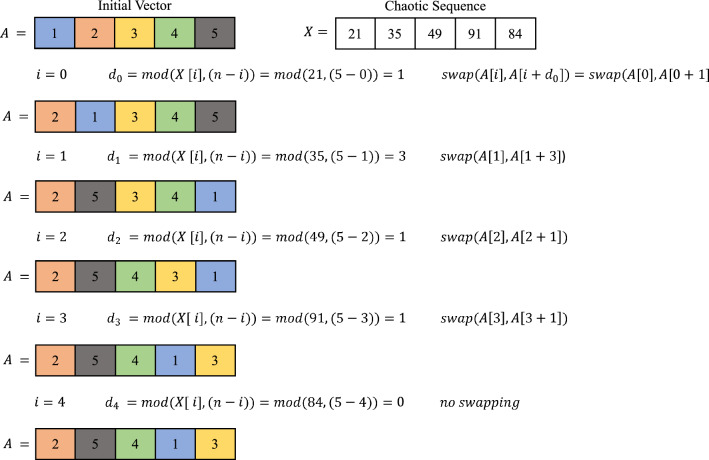
An example of the proposed Random Shuffle technique.

Based on this Fisher-Yates shuffle, we have proposed a Random Shuffle Technique (RST) using a chaotic sequence *X*. An example of RST is illustrated in Fig. 8. Let $A=[1,2,3,4,5]$ be the initial vector and $X=[21,35,49,91,84]$ be the chaotic sequence, and *n* is the number of elements in *X*. For each iteration, *i*, we calculate $d_i$ as

$$\begin{aligned}d_i=mod(X[i],(n-i))~~~~~\text {for~ } 0\le i \le n-1 \end{aligned}$$

Then swap the elements *A*[*i*] & $A[i+d_i]$. Each iteration *A* will be updated, and after the final step, we get $A=[2,5,4,1,3]$, which is the shuffled vector $A_{shuff}$. The proposed RST is algorithmically described in **Algorithm 1: RST()**.

**Algorithm 1:**
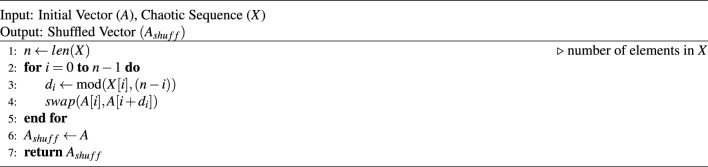
RST (*A*, *X*).

**Fig. 9:**
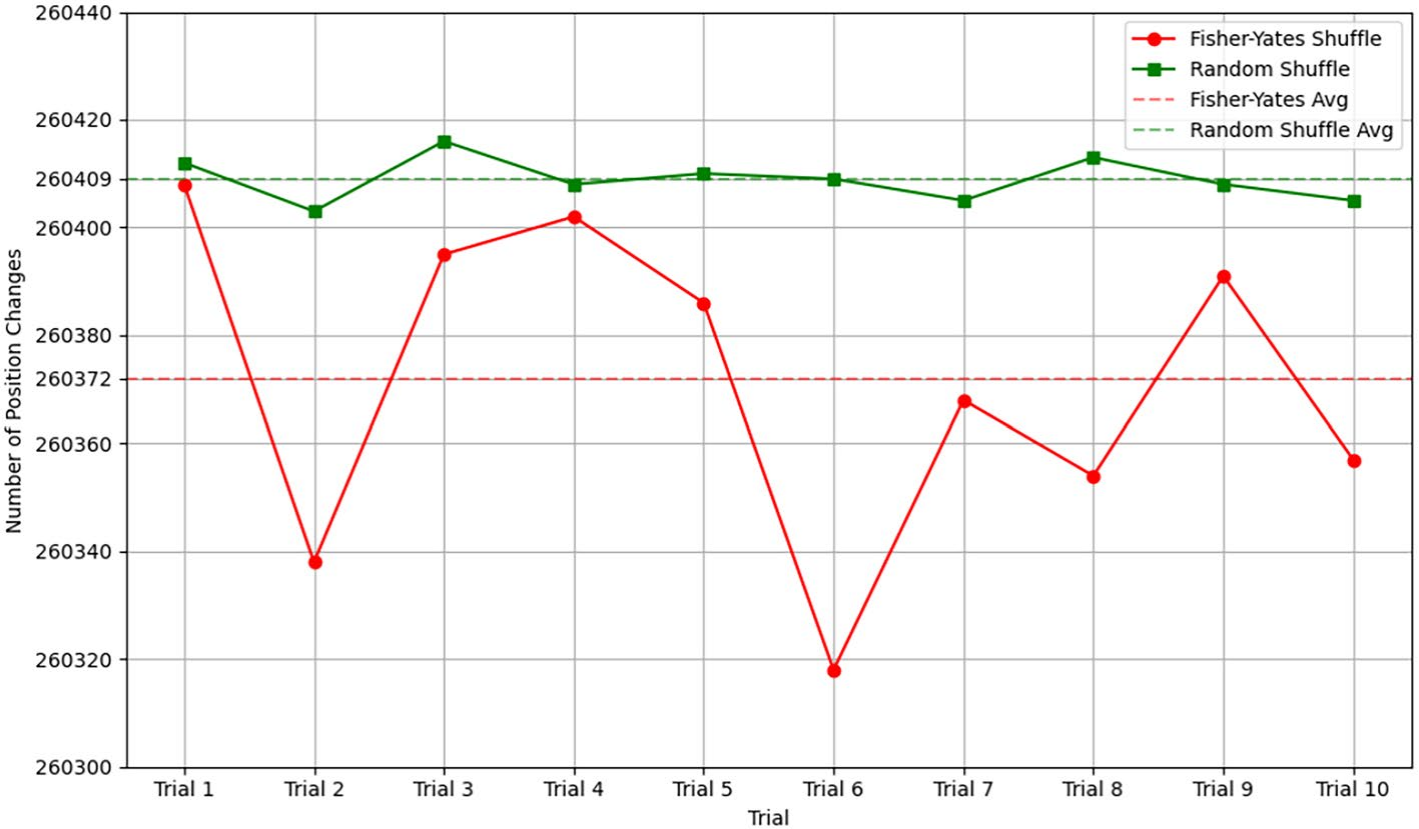
Comparative analysis of Fisher-Yates and RST based on position changes.

In principle, both FYS and RST algorithms are the same. The basic difference between these algorithms is that, in the FYS method, a random integer number is generated within [0, *i*], where the domain size is very limited. On the other hand, in the RST method, the sequence generated by 6DHCS is used; the domain of the generated numbers is much greater than that of the FYS method. Therefore, the shuffling rate (the number of positions changed after permutation) of the RST is higher. To evaluate the effectiveness and randomness of two permutation strategies, we consider ten different sets of numbers of size $2^{18}$. In this experiment, for each iteration of FYS, we change the ‘seed’ value, and for RST, we use different sequences generated by the proposed 6DHCS. As shown in Fig. 9, both methods consistently altered a large number of positions in the trials. The FYS resulted in an average of 260372 (99.32%) position changes, while RST achieved a slightly higher average of 260409 (99.34%). The closeness of these values indicates that both approaches highly permute the positions, whereas RST shows marginally more value across the trials. Hence, the proposed RST is suitable for use in different applications.

### Trifid cipher algorithm for image

**Fig. 10:**
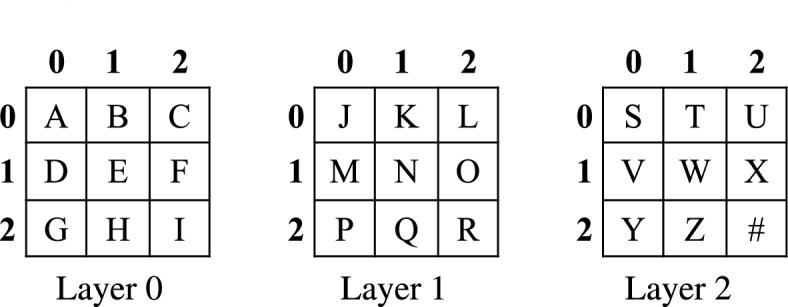
Example of Polybius cube for Trifid Cipher.

The Trifid Cipher, invented by Felix Delastelle in 1902^55^, is a classical cipher that extends the concept of the Bifid Cipher by introducing a third dimension. While the Bifid Cipher uses a two-dimensional Polybius square, the Trifid Cipher operates on a three-dimensional Polybius cube, enhancing both diffusion and confusion, which are two fundamental principles of cryptography. The Trifid Cipher encodes each letter using three coordinates: (i) *layer*, (ii) *row*, and (iii) *column*. Each alphabet letter is assigned to a position in a $3 \times 3 \times 3$ cube. Since there are 26 letters in the English alphabet, the remaining slot is filled with an auxiliary symbol (e.g., dot(.), $\#,+$, etc.). For example, we use a standard alphabet cube with mappings, as illustrated in Fig. 10.

**Table 6:** Triplet generation.

Coordinates	Letters										
	I	N	S	T	I	T	U	T	I	O	N
Layer	0	1	2	2	0	2	2	2	0	1	1
Row	2	1	0	0	2	0	0	0	2	1	1
Column	2	1	0	1	2	1	2	1	2	2	1

**Table 7:** Encoded message.

	Groups										
	012	202	220	112	100	200	021	121	012	121	221
Layer	0	2	2	1	1	2	0	1	0	1	2
Row	1	0	2	1	0	0	2	2	1	2	2
Column	2	2	0	2	0	0	1	1	2	1	1
Letters	F	U	Y	O	J	S	H	Q	F	Q	Z

For each letter in the message, we find its position in the cube and record the corresponding triplet (*layer*, *row*, *column*) in Table 6 based on the Polybius cube. It should be noted that, to represent a triplet, we need 6 bits (2 bits for each of *layer*, *row* and *column*). Suppose that the original message contains *n* letters; so according to the Trifid Cipher method, *n* triplets will be generated, which consume a total of 6*n* bits. In this cipher method, these triplets are concatenated into a single sequence (*Seq*) as follows:

$$\begin{aligned} Seq=layer_1~||~layer_2~||~\cdots ~layer_n~||~row_1~||~row_2~||~\cdots ~row_n~||~column_1~||~column_2~||~\cdots ~||~column_n \end{aligned}$$

In the cipher step, starting from the first number of *Seq*, three consecutive numbers are taken, and each number consumes 6 bits. Using these numbers, a new triplet is defined and this triplet helps to find a letter from the Polybius cube (as shown in Fig. 10). This letter will be used to replace the current letter in the original message. In the next iteration, the next three numbers of *Seq* will be used; this process continues while *Seq* is nonempty.

**Example 1:** To exemplify the encoding procedure using the Polybius cube (depicted in Fig. 10), we will examine the message ‘INSTITUTION’. Each character in the message is first assigned a triplet (layer, row, column) using the Polybius cube, with the associated triplets for all letters enumerated in Table 6. The triplets are transformed into a single sequence of digits by concatenation of all the values of the layer, row, and column, and yielding the sequence:

$$\begin{aligned} Seq=\underbrace{01220222011}_{layer}~\underbrace{21002000211}_{row}~\underbrace{21012121221}_{column}\end{aligned}$$

This sequence is divided into triplets, with each triplet containing three subsequent digits. There are 11 such triplets; this implies that the original message contains 11 letters. The partition of *Seq* is given below, where the groups are separated by a pair of parentheses.

$$\begin{aligned} Seq=(012)(202)(220)(112)(100)(200)(021)(121)(012)(121)(221)\end{aligned}$$

Each of these new triplets is subsequently mapped back to a letter with the Polybius cube, thus producing a new encoded character for each original one. The initial characters are substituted with these newly acquired letters to create the final encoded message. Table 7 illustrates that the message ‘INSTITUTION’ is converted into the ciphertext ‘FUYOJSHQFQZ’.

**Fig. 11:**
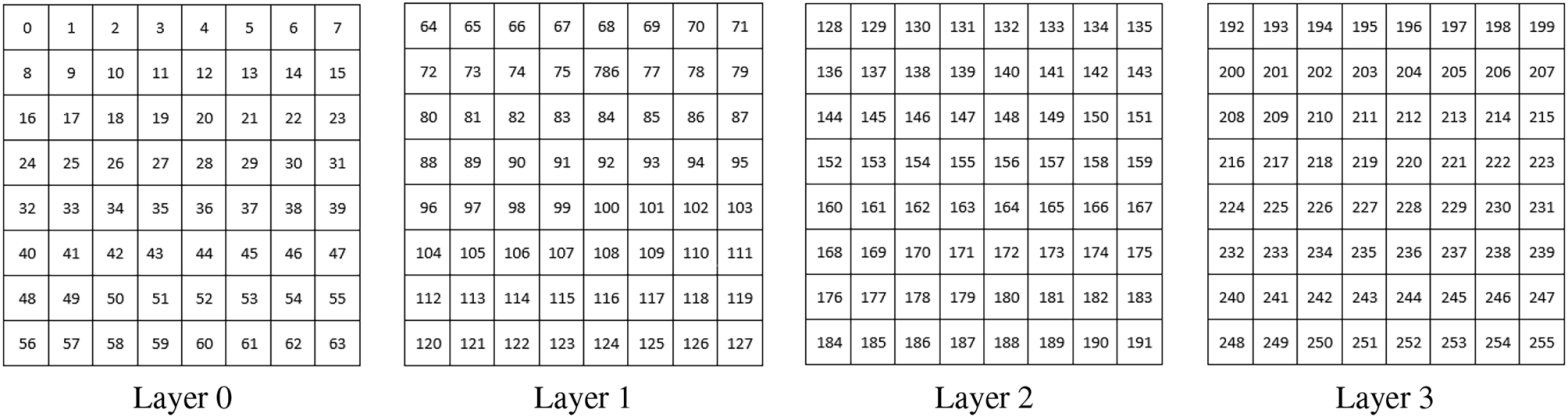
Trifid Cipher scheme for an 8-bit gray scale image.

Inspired by the classical Trifid cipher, we introduce a novel Trifid Cipher Algorithm for an image (TCAI). The Trifid Cipher method is defined to diffuse the intensity value of the pixels. A grayscale image has intensity values from 0 to 255 (for an 8-bit image). These 256 values are divided into four parts, each part having 64 values. So, we define a 3D matrix M of size $4\times 8 \times 8$, where the $i^{th}$ plane of M stores the value (*v*), $i*64 \le v < (i+1)*64$, sequentially in row-major order, for *i* = 0 to 3 (shown in Fig. 11). In this method, an intensity value $v~ (0 \le v \le 255)$ is encoded by a triplet (*l*, *r*, *c*) as given in Eq. (7), where $l, r, \text {and } c$ represents the *layer*, *row* and *column* as the basic Trifid Cipher.

7 $$\begin{aligned} (l, r, c)~=~ \text {Encode}(v);~~~~ \text {where}~~ l=(int)\frac{v}{64}; r=(int)\frac{v- l \times 64}{8}; ~\text {and}~ c=\text {mod}(v, 8) \end{aligned}$$

For example, consider the intensity value 123, which belongs to the position (7, 3) of *layer*1. Here, $l=(int)\frac{123}{64}=1$, $r=(int)\frac{123-1\times 64}{8}=(int)\frac{59}{8}=7$ and $c=\text {mod}(123,8)=3$.

It should be noted that the total bit requirement to represent the triplet (*l*, *r*, *c*) is 8 (since $0 \le l \le 3, 0 \le r, c \le 7$), which is the same as that of a pixel. For each pixel, $p_i$ the corresponding triplet $(l_i, r_i, c_i)$ is calculated and stored in three arrays *X*, *Y* and *Z*, respectively, as $X[i]=l_i, Y[i]=r_i$ and $Z[i]=c_i$.

These three arrays are then scrambled using chaotic sequences $X_1, X_2$ and $X_3$, producing the permuted arrays $X',Y'$ and $Z'$, respectively. Similar to the original Trifid Cipher method, we concatenate these three permuted arrays, and a new sequence *D* is defined as

8 $$\begin{aligned} D = \underbrace{X'}_{2n\text {-}bits} || \underbrace{Y'}_{3n\text {-}bits} ||\underbrace{Z'}_{3n\text {-}bits}, ~~~~n=P \times Q,~\text {number of pixels in the image} \end{aligned}$$

To intermix the encoded bit sequence with the neighbor pixel, we rotate *D* circularly by an amount not divisible by 8. Here, a 13-bit circular shift is applied, and $D'$ is obtained. Then, a bitwise XOR operation is performed between $D'$ and the chaotic sequence $X_4$, resulting in the sequence *E*, which is subsequently segmented into 8-bit chunks, resulting in $n=P\times Q$ such chunks. The $k^{th}$ chunk, $ch_k$, is partitioned into $l_k$, $r_k$ and $c_k$ where

9 $$\begin{aligned} ch_k=l_k||r_k||c_k \text {,~} |l_k|=2, |r_k|=3, \text { and } |c_k|=3. \end{aligned}$$

From the triplet $(l_k, r_k, c_k)$ using Eq. (10), we compute the value *p* and this value is stored in the (*i*, *j*)-th location of the diffused image $(img_{diff})$, where $i = (int)\frac{k}{Q}$ and *j* = mod(*k*, *Q*).

10 $$\begin{aligned} p=l_k\times 64+r_k\times 8+c_k \end{aligned}$$

The TCAI is algorithmically described in **Algorithm 2: TCAI()**.

**Algorithm 2:**
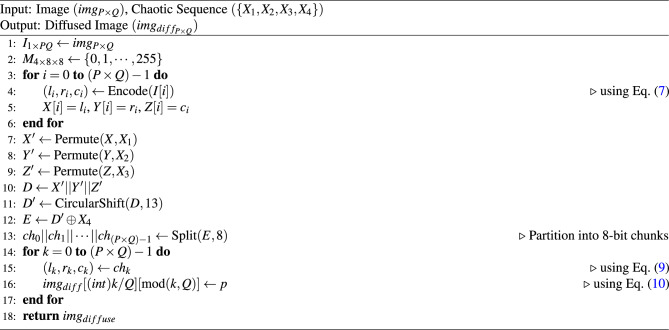
TCAI $$(img, \{X_1, X_2, X_3, X_4\})$$.

### Encryption process

**Fig. 12:**
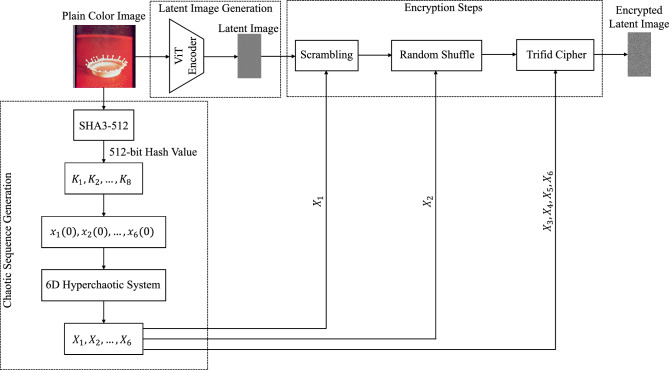
Proposed encryption process.

Fig. 12 illustrates the structural layout of the encryption procedure of the suggested method. The present method has three major parts: i) generating chaotic sequences, ii) deriving the latent image from the plain image, and iii) finally, the latent image is encrypted.

To generate chaotic sequences, we define 6DHCS, as discussed above, and the initial value of the state variables of 6DHCS is derived from the hash value (*H*) of the plain image. Here, the hash value *H* is generated by applying the SHA3-512 algorithm. The hash value *H* is partitioned into eight parts $K_i: 1 \le i \le 8$ such that each part has 64 bits, i.e.,

$$\begin{aligned} H~=~K_1||K_2||K_3||\cdots ||K_8,~\text {and }|K_i|=64 \end{aligned}$$

These components of *H* are then utilized to determine the initial value of the state variables of the 6DHCS using Eq. (11).

11 $$\begin{aligned} x_i(0)=((K_i\oplus K_{i+1}\oplus K_{i+2})/2^{68})+1, \quad \text {for} \quad 1\le i \le 6 \end{aligned}$$

Then, the proposed 6DHCS is executed iteratively with the above initial values and generates six sufficiently long chaotic sequences $\{X_1, X_2, X_3, X_4, X_5, X_6 \}$.

To achieve real-time encryption for large-sized color images, like SoA methods, we compress the original color image using our proposed ViTAE method, which encodes an input image into a latent image, and we achieve a compression ratio of 1.5:1. Unlike a standard ViT autoencoder, no masking is applied in the self-attention mechanism, as the entire sequence is processed in parallel for image reconstruction, resulting in excellent perceptual fidelity and structural preservation, as reported in the performance analysis of the suggested ViTAE.

In the final stage of the proposed encryption method, the latent image (*LI*) is scrambled (or permuted) using the sequence $X_1$. Then, the scrambled image is further scrambled using the proposed RST method (**Algorithm** 1**: RST()**), where the sequence $X_2$ is considered a parameter in the RST method. Furthermore, the latest version of the scrambled image is diffused by the proposed TCAI method (**Algorithm** 2: **TCAI()**), and we obtain our desired encrypted image, which retains the exact dimensions as the latent image. The proposed encryption method is outlined algorithmically in **Algorithm 3: Encryption()**.

**Algorithm 3:**
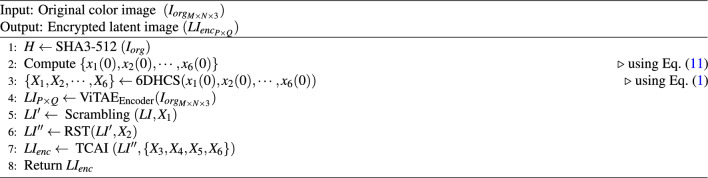
Encryption $$(I_{org}, LI_{enc})$$.

### Decryption process

In symmetric encryption, the original data is precisely reconstructed by reversing the encryption procedures using the same secret key during the decryption phase. In this context, the encrypted latent image $LI_{enc}$ is decrypted by applying the inverse of the encryption steps InvTCAI(), InvRST(), and InvScrambling() sequentially, using the same chaotic sequences $\{X_1, X_2, \cdots , X_6\}$ generated by 6DHCS with the same initial values derived from the same hash value *H*, which is the secret key of the proposed method. This ensures the accurate recovery of the latent image *LI*. The decrypted latent image $LI_{d}$ is then passed through the decoder of the ViTAE model to reconstruct the final reconstructed image $I_{dec}$. Although the method is inherently lossy due to latent representation and compression, the reconstructed image $I_{dec}$ remains visually and structurally similar to the original image $I_{org}$. To assess the perceptual quality of the reconstructed images, we use several metrics such as PSNR, SSIM, MS-SSIM, FSIM, and VIFP as discussed above.

**Algorithm 4:**
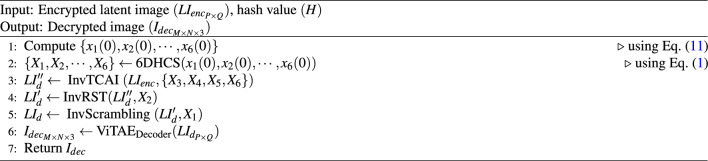
Decryption $$(LI_{enc}, H, I_{dec})$$.

## Experimental results and analysis

**Fig. 13:**
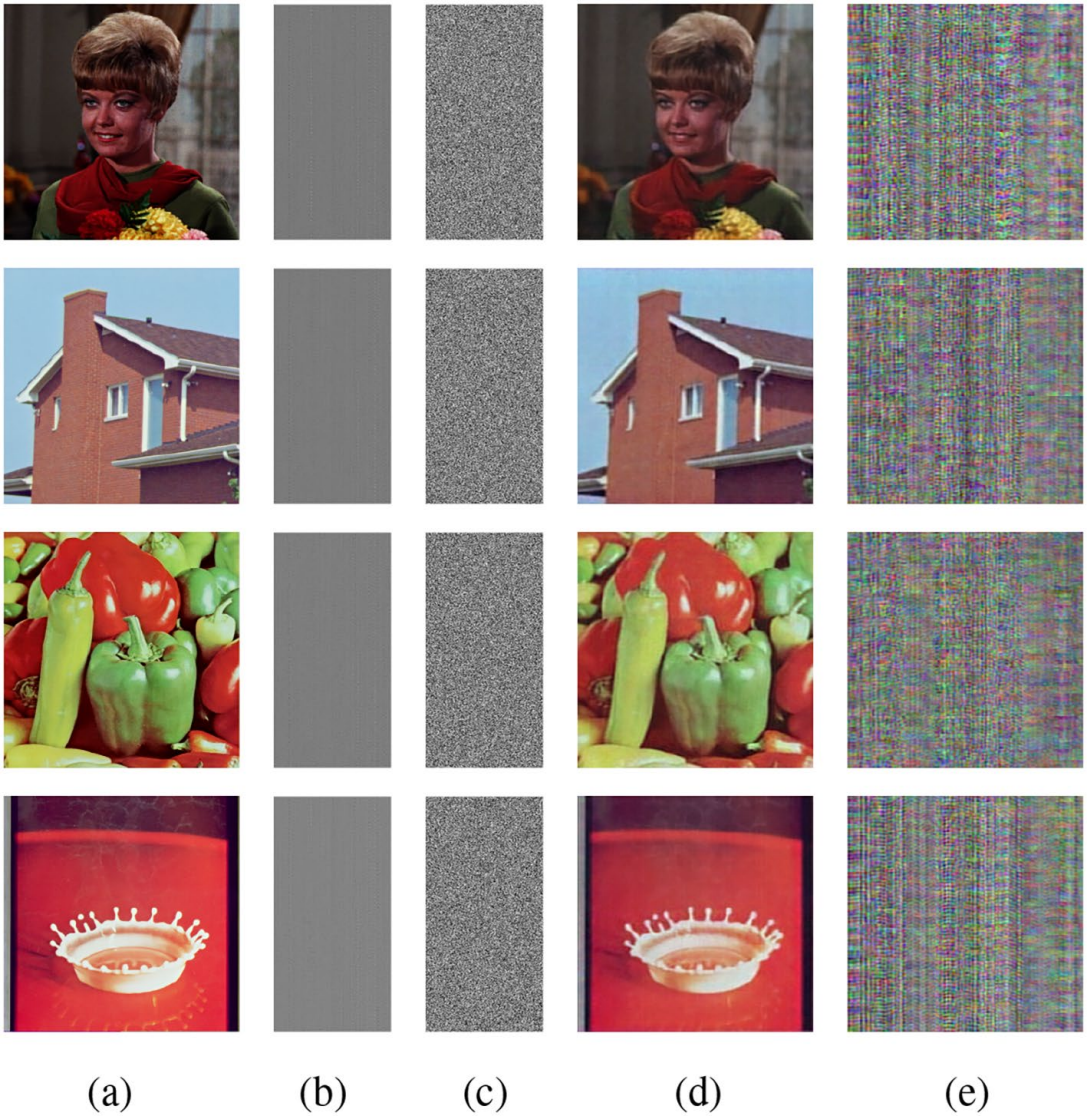
(**a**) Original image, (**b**) latent image, (**c**) encrypted latent image, (**d**) decrypted image, (**e**) decrypted image with wrong key.

The present approach is carried out on a platform with an Intel(R) Core(TM) i5-10300H processor running at 2.50 GHz, 8 GB of RAM, a 64-bit Windows operating system, and Python version 3.11.6. Furthermore, various color images with $256\times 256$ sizes are selected from the USC-SIPI database^56^ for experimental evaluations. Fig. 13 depicts the outcomes of the encryption and decryption process of the suggested technique. The test results unequivocally illustrate the efficacy of the suggested encryption algorithm in encrypting images. The encrypted images exhibit a noise-like pattern, ensuring that no discernible information about the original plain images is apparent. Upon decryption, the recovered images closely resemble the plain images, while using a slightly modified key fails to retrieve the original content. This highlights the algorithm’s ability to achieve secure transmission through robust encryption and decryption processes. Multiple performance metrics are used to evaluate the efficacy of the suggested method. These include key space analysis, entropy analysis, histogram analysis, polar histogram analysis, $\chi ^2$ test, correlation analysis, sensitivity to plaintext and key variations, defence against differential, cropping, and noise attacks, and analysis of computational complexity, etc.

### Key space analysis

To safeguard against brute-force attacks, an effective image encryption method must have a large key space. A cryptographic system is considered secure if its key space is at least $2^{100}$^57^. In our approach, the SHA3-512 algorithm is employed to generate a 512-bit hash value *H* from the plain image. This hash *H* is used to initialize the state variables of the 6DHCS, which controls the behavior of the encryption process. As the suggested method is symmetric, *H* serves as the private key. Consequently, the key space of the proposed method is determined using 512 bits. Given that $2^{512} \gg 2^{100}$, the proposed scheme offers strong resistance to brute-force attacks and ensures robust cryptographic security.

### Entropy analysis

Entropy plays an important role in assessing the inherent unpredictability and randomness of an image. As the pixel value distribution in an original image is typically non-uniform, a robust encryption algorithm should strive to generate a uniform pixel distribution. A more uniform distribution signifies higher entropy, reflecting increased randomness and security. The entropy is computed using Eq. (12).

12 $$\begin{aligned} H(x)=\sum _{i=0}^{2^{n-1}}p(x_i)\log _2\Big (\frac{1}{p(x_i)}\Big ) \end{aligned}$$

where *x* is the pixel value, $p(x_i)$ is the probability of $x_i$, and $2^n$ is the general states.

Achieving the maximum entropy value of 8 (for an 8-bit image) is essential for enhancing resistance to statistical attacks. The effectiveness of an encryption algorithm is closely associated with attaining an entropy value close to 8, which signifies a high level of randomness and security^58^. As detailed in Table 8, a key finding is that our approach achieves an entropy value close to 8. This notable result reveals a high degree of unpredictability in the cipher latent image generated by the suggested technique. The higher unpredictability means that deciphering the original image from its encrypted version becomes more challenging for attackers. Table 9 compares the suggested technique with SoA methods. It may be noted that our method achieves an average entropy of 7.9986, which is aligned with the ideal value and comparable to the SoA methods. This establishes the effectiveness of our approach.

**Table 8:** Entropy analysis of original and encrypted latent images.

Image	Original Latent Image	Encrypted Latent Image
Barbara	4.7431	7.9987
Boats	4.6252	7.9984
Female	4.5984	7.9987
House	4.5192	7.9986
Jelly beans	4.4527	7.9985
Peppers	4.7196	7.9984
Plane	4.5989	7.9986
Sailboat	4.8262	7.9985
Splash	4.6805	7.9985
Tree	4.9068	7.9986
Average	4.6671	7.9986

**Table 9:** Entropy analysis comparison.

Method	Entropy
In^23^	7.9983
In^24^	7.9937
In^25^	7.9970
In^26^	7.9976
In^30^	7.9770
In^36^	7.9749
In^37^	7.9973
Proposed	7.9986

### Local shannon entropy analysis

Local Shannon entropy provides an alternative mechanism for evaluating the randomness within a message. While the overall (global) intensity distribution of an image might appear nearly uniform, certain areas may still exhibit non-uniform local distributions. These local inconsistencies can potentially lead to misleading conclusions about the actual unpredictability or randomness of the image content. To address this issue, local Shannon entropy is employed as a complement to the conventional global entropy measure, offering a more detailed assessment of randomness in specific regions of the image^59^. The local entropy is represented as:

13 $$\begin{aligned} \overline{H_{k,T_B}}=\frac{1}{k}\sum _{i=1}^{k}{H(S_i)} \end{aligned}$$

Here, *k* represents the number of non-overlapping local blocks of equal size within the image, $S_i$ denotes the $i^{th}$ local block where $i \in [1, k], H(S_i)$ is the entropy of block $S_i$, and $T_B$ is the number of pixels contained in each block. Following the SoA approach, this analysis adopts $k = 30$ and $T_B = 1936$. At a 0.05 significance level, the optimal range for local entropy is between 7.901901305 and 7.903037329^59^. Table 10 presents the local entropy values for the encrypted latent images under these parameters. As illustrated in the table, the proposed method successfully meets the required criteria for all encrypted outputs.

**Table 10:** Local Shannon entropy value of different encrypted latent images.

Image	Local Shannon entropy	Remark
Barbara	7.90271	$\checkmark$
Boats	7.90256	$\checkmark$
Female	7.90268	$\checkmark$
House	7.90232	$\checkmark$
Jelly beans	7.90205	$\checkmark$
Peppers	7.90302	$\checkmark$
Plane	7.90240	$\checkmark$
Sailboat	7.90281	$\checkmark$
Splash	7.90249	$\checkmark$
Tree	7.90243	$\checkmark$

### Histogram analysis

**Fig. 14:**
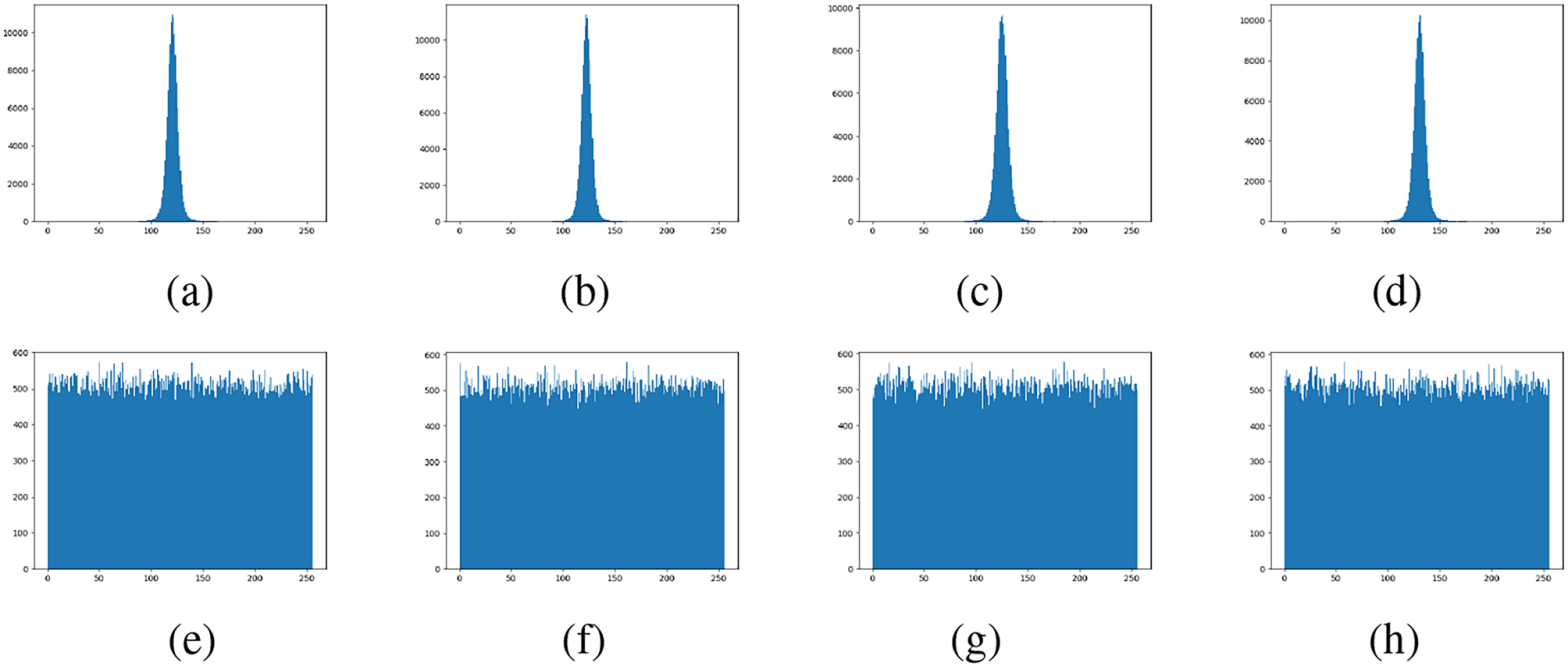
Histogram analysis: (**a**) - (**d**) Histograms of original latent images, (**e**) - (**h**) histograms of encrypted latent images.

The histogram test evaluates the uniformity of intensity values in cipher images, a key indicator of an algorithm’s resistance to statistical attacks^60^. Fig. 14 depicts the histograms of the latent image of ‘Female’, ‘House’, ‘Peppers’, and ‘Splash’ alongside those of their encrypted latent image. The cipher latent images exhibit uniform and consistent histograms, unlike the original latent images, which display nonuniform and distinct distributions. This indicates that the encryption process effectively randomizes pixel intensity values, thereby enhancing the system’s resistance to statistical attacks.

### Polar histogram analysis

The polar histogram is employed to evaluate the statistical behaviour of pixel intensity distributions in both the plain and encrypted latent images. It provides an intuitive visualization by mapping the intensity levels (0–255) along the angular axis and their corresponding normalized frequencies along the radial direction. This representation facilitates a comparative assessment of the uniformity and randomness characteristics of the image data before and after encryption^61^.

In the plain image, the polar histogram generally exhibits smooth fluctuations with noticeable peaks corresponding to dominant intensity values. These peaks reflect the presence of homogeneous brightness regions or repetitive texture patterns, which indicate structural regularities inherent to the original image content. Conversely, the encrypted latent image shows a polar histogram with a markedly uniform distribution of intensities. The absence of peaks and the more uniformly distributed radial pattern ensure that the encryption process has successfully disrupted the statistical correlations of the plain latent image. This uniformity suggests that the proposed encryption algorithm effectively randomises pixel intensities, thereby concealing visual patterns and minimising redundancy.

**Fig. 15:**
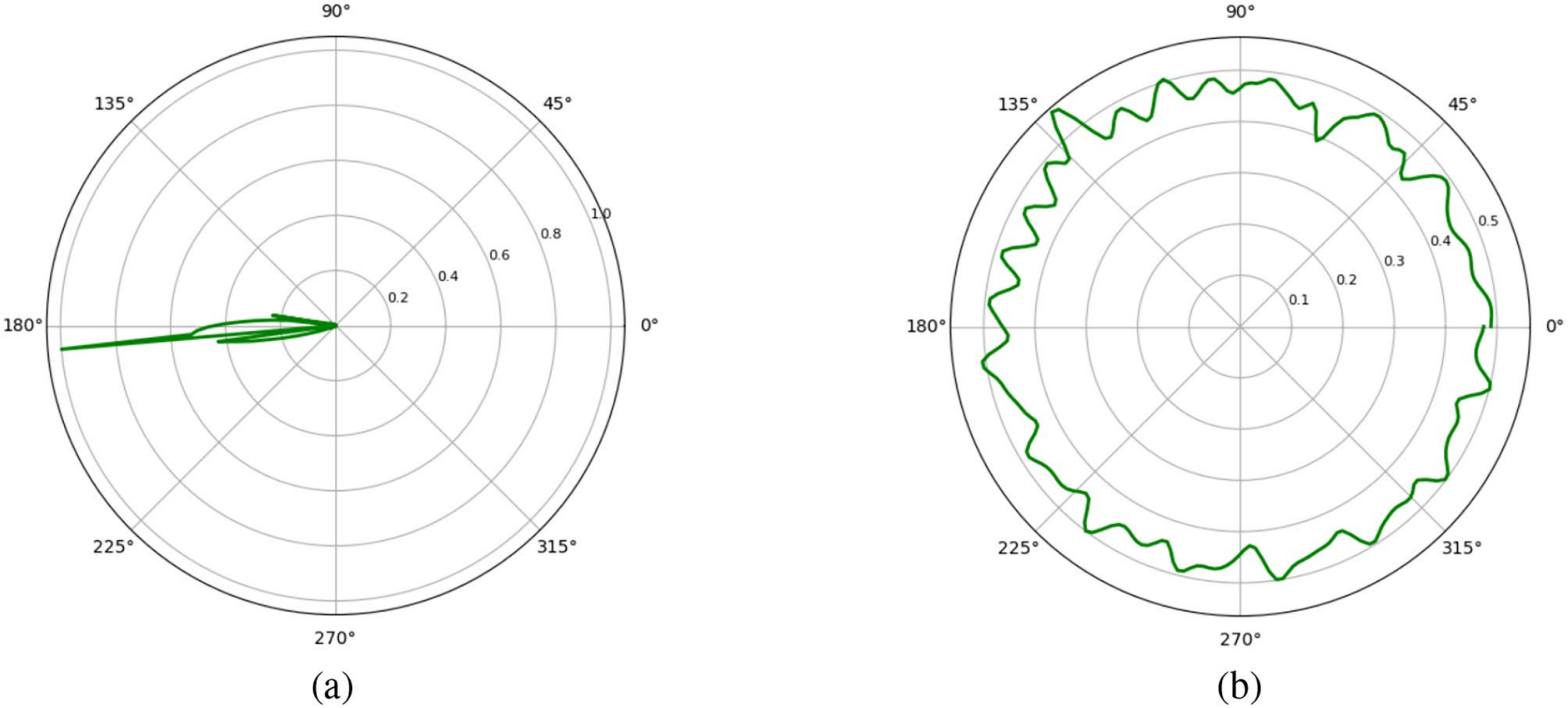
Polar histogram analysis: (**a**) original latent image, (**b**) encrypted latent image.

As illustrated in Fig. 15, the polar histogram of the encrypted latent ‘Splash’ image (Fig. 15(b)) shows a consistent, evenly distributed circular pattern compared to the plain latent image (Fig. 15(a)), which contains distinct peaks and variations. This outcome validates the ability of the proposed scheme to achieve strong statistical diffusion and enhance resistance against histogram-based cryptanalytic attacks.

### $\chi ^2$ Test

The Chi-square ($\chi ^2$) test is employed as a statistical measure to evaluate the uniformity of a histogram quantitatively. The $\chi ^2$ statistic is calculated using Eq. (14).

14 $$\begin{aligned} \chi ^2=\sum _{i=0}^{255}\frac{(O_i-E)^2}{E} \end{aligned}$$

where, $O_i$ denotes the observed frequency of pixel intensity *i*, and *E* represents the expected frequency for each intensity level in a perfectly uniform image, calculated as $E = \frac{M \times N}{2^k}$ for an 8-bit image $(k = 8)$. In this context, $M\times N$ is the size of the image, and $2^k (= 256)$ indicates the total number of gray levels.

The $\chi ^2$ result for the encrypted latent images is depicted in Table 11. Notably, all the computed values fall below the critical threshold of $\chi ^2(0.05, 255) = 293.2478$^62^, confirming that the encrypted latent images maintain uniformly distributed histograms. This result validates the effectiveness of the suggested technique in resisting statistical attacks.

**Table 11:** Chi-square test results for encrypted latent images.

Image	Chi-square Value	Remark
Barbara	229.7031	$\checkmark$
Boats	286.0117	$\checkmark$
Female	239.8633	$\checkmark$
House	263.5781	$\checkmark$
Jelly beans	274.7031	$\checkmark$
Peppers	291.3594	$\checkmark$
Plane	254.6211	$\checkmark$
Sailboat	278.9102	$\checkmark$
Splash	282.1172	$\checkmark$
Tree	250.8398	$\checkmark$

### Correlation analysis

**Fig. 16:**
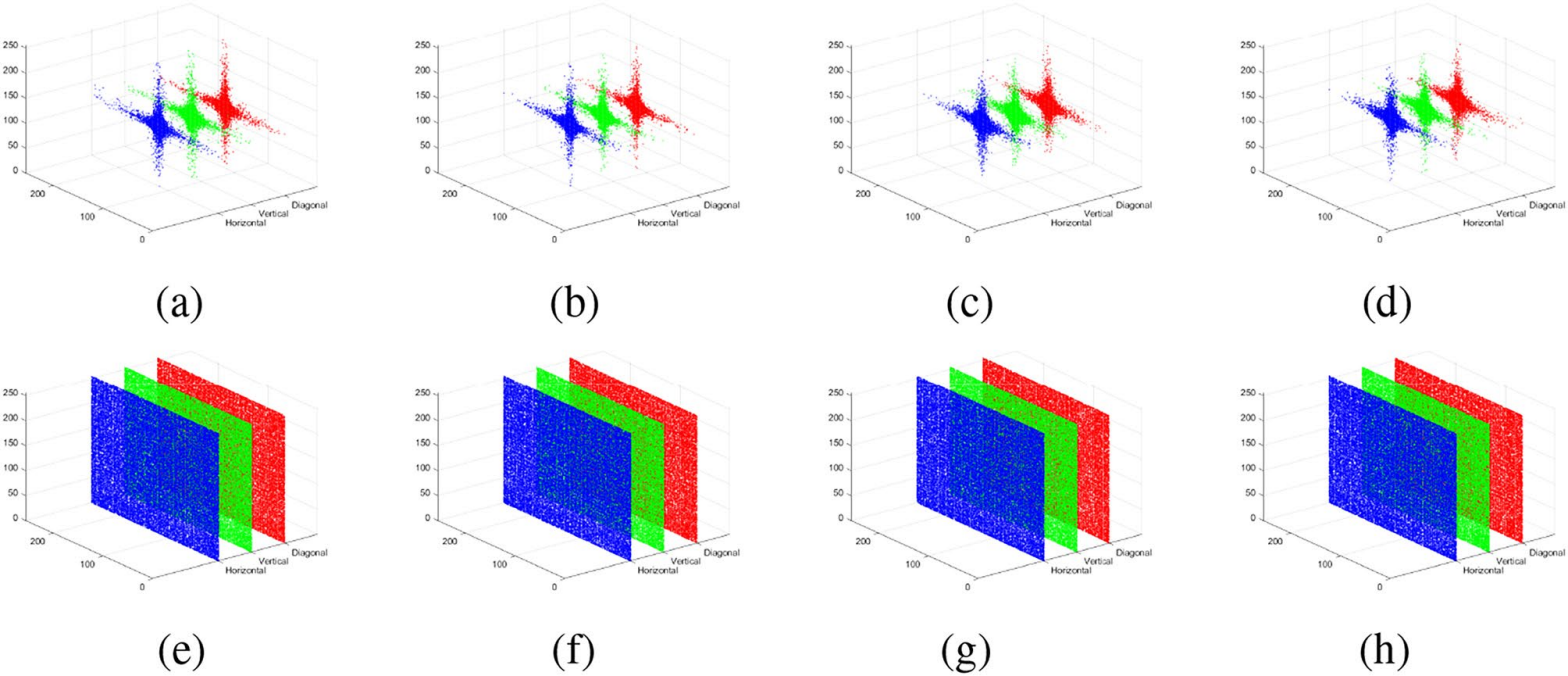
Correlation analysis: (**a**) & (**c**) Correlation of original latent image, (**b**) & (**d**) Correlation of encrypted latent image.

A reliable image encryption technique should minimize the correlation among adjacent pixels to defend against statistical assaults. In this work, the correlation in the encrypted latent images is evaluated along the horizontal (H), vertical (V), and diagonal (D) directions using 10,000 randomly selected pairs of neighboring pixels. Fig. 16 illustrates the scatter plots for the ‘Female’, ‘House’, ‘Peppers’, and ‘Splash’ images across these three orientations. As observed, the original latent images exhibit a strong correlation. In contrast, the encrypted latent images show a substantial decrease in correlation, with pixel pairs widely dispersed throughout the entire area. These results demonstrate that the suggested encryption technique significantly disrupts pixel relationships, offering strong protection against statistical analysis.

The correlation coefficient is a statistical measure used to assess the strength and direction of a linear relationship between two variables. Its value ranges from -1 to 1, where -1 indicates a perfect negative correlation, 1 indicates a perfect positive correlation, and 0 denotes the absence of a linear relationship. The correlation coefficient $(r_{\beta \gamma })$ is computed using Eq. (15):

15 $$\begin{aligned} \begin{aligned} r_{\beta \gamma }=\frac{\displaystyle \sum _{i=1}^N (\beta _i-E(\beta ))(\gamma _i-E(\gamma ))}{\sqrt{\displaystyle \sum _{i=1}^N (\beta _i-E(\beta ))^2} \sqrt{\displaystyle \sum _{i=1}^N (\gamma _i-E(\gamma ))^2}}\\ \end{aligned} \end{aligned}$$

where $(\beta _i, \gamma _i)$ represent the intensity values of the $i^{th}$ selected pair, *N* denotes the total number of pixel pairs, and $E(\beta )$ and $E(\gamma )$ are the mean values of $\{\beta _i\}$ and $\{\gamma _i\}$, respectively. Table 12 summarizes the correlation results for various test images, which show a consistently low correlation among the encrypted pixels. This low correlation demonstrates the ability of the proposed scheme to disrupt predictable pixel relationships. Furthermore, Table 13 presents a comparison of the correlation coefficients obtained using the proposed method with SoA techniques. The comparison reveals that the proposed method achieves competitive performance in minimizing adjacent pixel correlation, thereby enhancing resilience against statistical attacks.

**Table 12:** Correlation analysis of original and encrypted latent images.

Image	Original Latent Image			Encrypted Latent Image		
	H	V	D	H	V	D
Barbara	0.011835	-0.011285	-0.010896	0.000023	-0.000011	0.000014
Boats	0.019781	-0.016129	0.010239	-0.000018	-0.000001	0.000017
Female	0.015792	-0.012788	-0.011746	-0.000011	-0.000006	-0.000037
House	0.011487	0.013535	0.011738	0.000045	0.000008	-0.000026
Jelly beans	0.015753	0.011330	0.013323	-0.000046	0.000009	-0.000034
Peppers	0.010959	0.010452	0.015101	0.000088	-0.000100	-0.000044
Plane	0.013108	-0.012208	-0.012572	0.000049	-0.000081	0.000063
Sailboat	-0.010525	0.011113	-0.010859	-0.000038	-0.000052	-0.000045
Splash	0.011349	0.016655	-0.010229	-0.000088	0.000002	-0.000101
Tree	0.011323	0.010619	0.013246	-0.000046	-0.000021	0.000068
Average	0.013191	0.012611	0.011995	0.000045	0.000029	0.000045

**Table 13:** Correlation analysis comparison.

Method	Direction	Correlation
In^23^	H	0.006200
	V	0.000305
	D	0.009750
In^24^	H	0.012533
	V	0.027833
	D	0.008800
In^25^	H	0.007300
	V	0.006025
	D	0.013175
In^26^	H	0.013800
	V	0.007733
	D	0.007600
In^30^	H	0.040225
	V	0.058400
	D	0.011025
In^36^	H	0.000500
	V	0.000660
	D	0.000220
In^37^	H	0.001175
	V	0.001925
	D	0.005950
Proposed	H	0.000045
	V	0.000029
	D	0.000045

### Plaintext sensitivity analysis

The proposed encryption scheme exhibits high plaintext sensitivity, meaning that altering just one bit of the plaintext results in a markedly different ciphertext. This is achieved by recalculating the hash value after altering the bit, which in turn alters the initial values of the state variables of the 6DHCS. Consequently, the 6DHCS generates entirely different sequences, resulting in different encrypted outputs.

To quantify this sensitivity, two widely used metrics, Number of Pixels Change Rate (NPCR), and Unified Average Changing Intensity (UACI), are used. These metrics are calculated using Eq. (16):

16 $$\begin{aligned} {\begin{matrix} NPCR & = \frac{1}{M \times N} \sum _{\beta =1}^{M} \sum _{\gamma =1}^{N} D(\beta ,\gamma ) \times 100 \% \\ UACI & = \frac{1}{M \times N} \sum _{\beta =1}^{M} \sum _{\gamma =1}^{N} \frac{|Enc_1(\beta ,\gamma ) - Enc_2(\beta ,\gamma )|}{255} \times 100 \% \end{matrix}} \end{aligned}$$

Here, $D(\beta ,\gamma )$ is expressed as:

$$\begin{aligned} D(\beta ,\gamma ) = {\left\{ \begin{array}{ll} 0 & \text {if } Enc_1(\beta ,\gamma ) = Enc_2(\beta ,\gamma ) \\ 1 & \text {if } Enc_1(\beta ,\gamma ) \ne Enc_2(\beta ,\gamma ) \end{array}\right. } \end{aligned}$$

In the above, $M\times N$ represents the size of the image. $Enc_1$ and $Enc_2$ denote the encrypted versions of the original and modified images. The ideal values for NPCR and UACI are $99.6094\%$ and $33.4635\%$, respectively^63^.

In our analysis, we randomly change one pixel in the plain image and compute the NPCR and UACI values between the two encrypted latent images that arise. Each image goes through this process ten times, and Table 14 displays the average outcomes. The obtained values are consistent with the theoretical benchmarks, validating the high plaintext sensitivity of the proposed algorithm.

**Table 14:** Plaintext sensitivity analysis.

Image	NPCR	UACI
Barbara	99.6131	33.4606
Boats	99.6124	33.4709
Female	99.6116	33.4519
House	99.6094	33.4467
Jelly beans	99.6154	33.4678
Peppers	99.6078	33.4774
Plane	99.6093	33.4621
Sailboat	99.6147	33.4600
Splash	99.6124	33.4644
Tree	99.6116	33.4547
Average	99.6118	33.4616

### Key sensitivity analysis

**Fig. 17:**
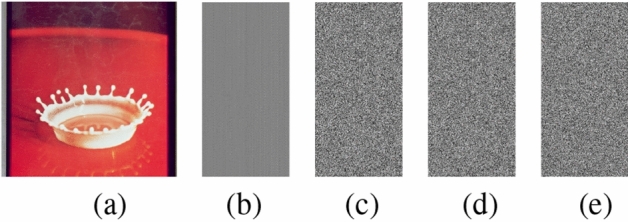
(**a**) Original image, (**b**) latent image, (**c**) encrypted latent image with original key, (**d**) encrypted latent image with wrong key, (**e**) difference encrypted latent image between (**c**) & (**d**).

The encryption and decryption procedures of the algorithm should be highly sensitive to key variations. Even a minor change in the key results in substantial differences in the encrypted and decrypted outputs. We experimented with using the latent ‘Splash’ image to assess the key sensitivity of the presented encryption technique. The latent image undergoes two separate encryption processes: one using the original key and the other using a slightly modified key created by flipping a single random bit in the original key. The corresponding encrypted latent images and their absolute difference are depicted in Fig. 17. In an ideal scenario, the absolute difference between two identical images would make an entirely black image, representing zero-valued pixels throughout. However, as shown in Fig. 17(e), a large number of pixels exhibit nonzero values, reflecting significant differences. The proportion of these non-zero pixels corresponds to the NPCR, which is presented in Table 15. The observed NPCR value of 99.6103% confirms that a minimal alteration in the key leads to an almost entirely different cipher latent image. This highlights the strong key sensitivity exhibited by the proposed method.

**Table 15:** Key sensitivity analysis.

Image	NPCR	UACI
Barbara	99.6109	33.4514
Boats	99.6162	33.4641
Female	99.6093	33.4608
House	99.6080	33.4661
Jelly beans	99.6071	33.4562
Peppers	99.6109	33.4596
Plane	99.6025	33.4509
Sailboat	99.6086	33.4838
Splash	99.6170	33.4656
Tree	99.6124	33.4671
Average	99.6103	33.4626

**Table 16:** Key sensitivity analysis comparison.

Method	NPCR	UACI
In^25^	99.5700	-
In^36^	99.5453	33.4224
Proposed	99.6103	33.4626

We conducted experiments by flipping different bits of the original key for each test image. NPCR and UACI assess the key sensitivity. The results in Table 15 indicate that the proposed method consistently achieves values very close to the ideal benchmarks for both NPCR and UACI. Furthermore, a comparative analysis shown in Table 16 highlights that our approach outperforms several existing methods regarding key sensitivity. Importantly, we also observed that the decryption process is sensitive to the correctness of the key. When an incorrect key is used for decryption, the output is a noise-like image with no meaningful visual content, as shown in Fig. 13(e). These findings confirm that the encryption and decryption processes of the proposed method are key sensitive, strengthening its robustness and security.

### Differential attack

In a differential attack, the assaulter attempts to extrapolate the secret key by making slight modifications to the original image and analyzing the differences between the resulting encrypted outputs. An effective encryption scheme should be highly sensitive to such minor changes in the plaintext to resist this form of attack. The proposed method is highly plaintext sensitive (refer to Sub-section Plaintext Sensitivity Analysis), ensuring that even a slight alteration in the input image generates a significantly different encrypted image. As a result, the suggested approach is well-equipped to defend against differential attacks. A comparative analysis with other SoA methods, as reported in Table 17, reaffirms the robustness of the proposed approach against this attacks.

**Table 17:** NPCR and UACI comparison.

Metric	Method					
	In^23^	In^24^	In^25^	In^26^	In^37^	Proposed
NPCR	99.6070	99.6338	99.6400	99.6073	99.6044	99.6118
UACI	33.4548	33.5452	33.5825	33.4558	33.4745	33.4616

### Cropping attack

Encrypted images may lose data while being sent. The receiver should be able to recognize the decrypted version even if certain parts of the ciphertext are lost. In this evaluation, selected portions of the cipher image are removed, and the resulting reconstructed image is assessed. This method is termed a cropping attack. In this test, certain regions of the cipher latent image are cropped and substituted with black pixels. The objective is to assess whether the decryption process can recover an image that closely resembles the original, even with incomplete data. The PSNR can assess the similarity between the plain image and the decrypted image. The PSNR value is computed using Eq. (17)

17 $$\begin{aligned} \begin{array}{l} PSNR=10\log _{10}\Big (\frac{MAX^2}{MSE}\Big ) (dB)\\ \\ MSE=\displaystyle \frac{1}{M\times N}\sum _{\beta =1}^{M}\sum _{\gamma =1}^{N}((I_{org}(\beta ,\gamma )-I_{dec}(\beta ,\gamma ))^2 \end{array} \end{aligned}$$

where $M\times N$ defines the image size, MAX stands for the peak pixel intensity, and $I_{org}$ and $I_{dec}$ denote the original and decrypted image, respectively.

**Fig. 18:**
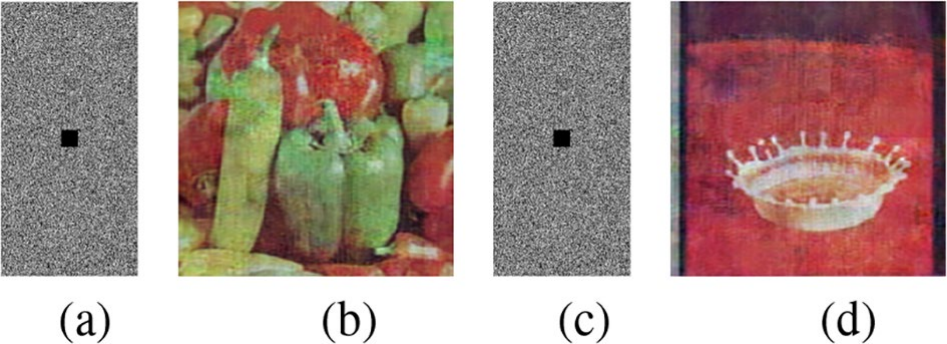
Cropping attack: (**a**) & (**c**) encrypted latent image with $32\times 32$ data loss, (**b**) & (**d**) decrypted image of (**a**) & (**c**), respectively.

Cropping attacks on ‘Peppers’ and ‘Splash’ are illustrated in Fig. 18. Here, we consider a block of size $32 \times 32$ that is cropped and show the reconstructed images due to data loss. Table 18 shows the PSNR scores for individual color planes of the decrypted images after cropping attacks, highlighting the effectiveness of the proposed method in handling such attacks.

**Table 18:** PSNR values of deciphered images under cropping attack.

Image	R	G	B
Barbara	14.8481	19.8999	11.9194
Boats	13.2059	17.4841	18.2715
Female	12.1673	16.2628	17.6424
House	13.2686	23.0980	13.8677
Jelly beans	15.0738	23.1560	13.5572
Peppers	8.3842	18.2785	11.4065
Plane	16.4982	19.8695	19.3492
Sailboat	12.9478	18.3209	18.4268
Splash	8.1414	18.4787	8.4416
Tree	16.7289	18.4562	16.1233
Average	13.1264	19.3305	14.9006

### Noise attack

**Fig. 19:**
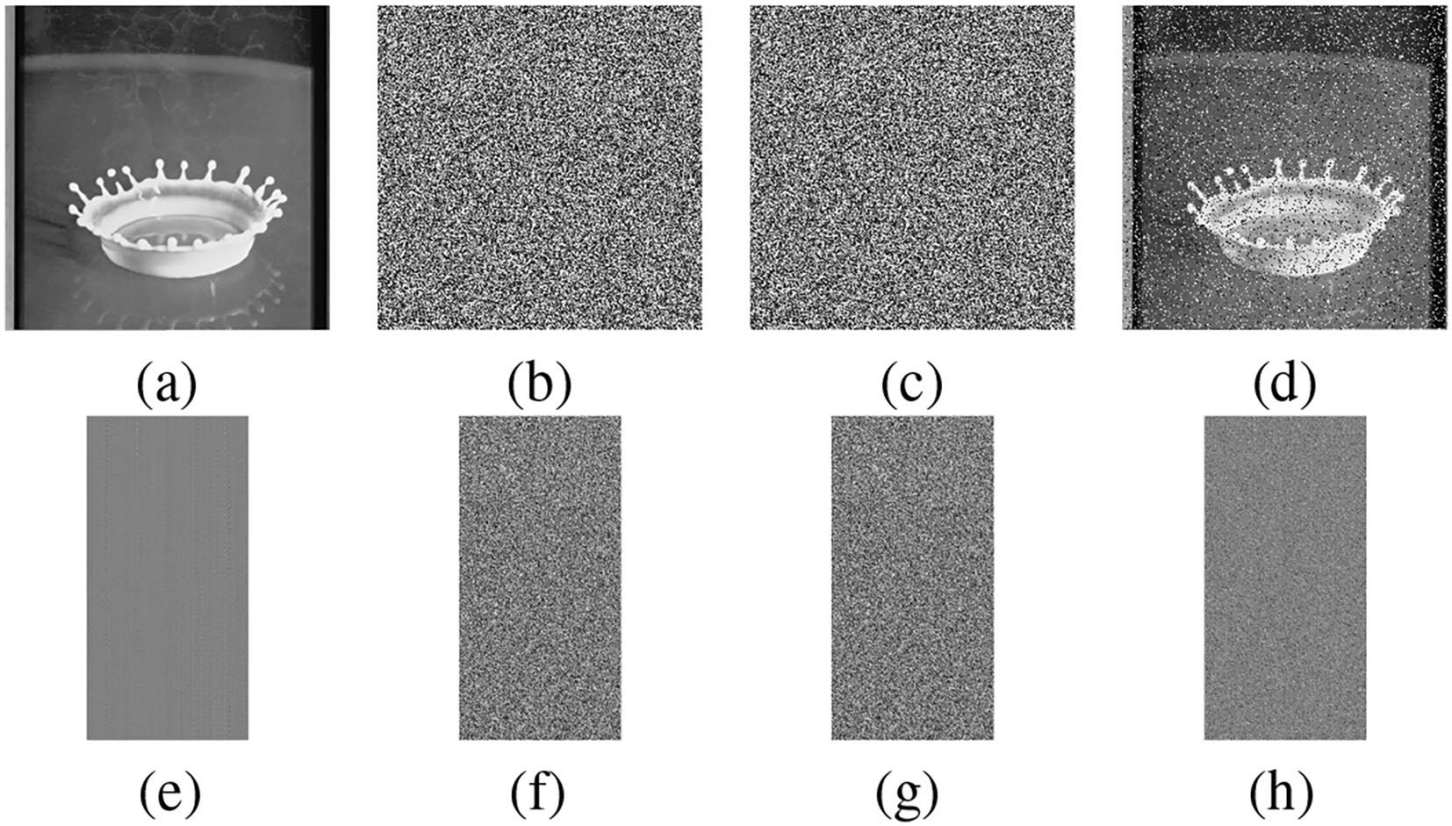
Noise attack analysis: (**a**) original image, (**b**) encrypted image (PSNR=8.7439), (**c**) encrypted image with salt & pepper noise (PSNR=8.7420) (**d**) decrypted image (PSNR=15.5592), (**e**) original latent image, (**f**) encrypted latent image (PSNR=10.6521), (**g**) encrypted latent image with salt & pepper noise (PSNR=10.6459), (**h**) decrypted latent image (PSNR=18.4016).

A robust image encryption technique should be capable of handling noise introduced during transmission, ensuring that the decrypted image remains recognizable even when the encrypted data is partially corrupted. The ViTAE model generates an abstract and highly compressed latent representation, where detailed spatial structures are transformed into high-level semantic features. This transformation inherently increases sensitivity to noise, as even small perturbations can disrupt the encoded feature distribution, making pixel-level reconstruction challenging.

To evaluate the robustness of the proposed method under such conditions, we tested its performance against salt & pepper noise and Gaussian noise. The experimental results indicate that the proposed scheme performs reasonably well when salt & pepper noise is applied to the spatially encrypted image. As shown in Fig. 19, the decrypted output corresponding to the noisy encrypted image remains visually identifiable, achieving a PSNR of 15.5592 relative to the original image. In the latent domain, applying the same level of salt & pepper noise to the encrypted latent representation still allows partial reconstruction, resulting in a PSNR of 18.4016 when compared to the original latent image.

However, under Gaussian noise attacks, the proposed method completely fails to resist the disturbance, indicating that the framework is not robust against such noise. Consequently, although the proposed encryption framework demonstrates partial robustness to noise–particularly in the spatially encrypted domain–it lacks sufficient resilience when noise is introduced into the latent encrypted representation.

### Time complexity analysis

The computational complexity is analyzed to ensure the suitability of the proposed encryption framework for real-time applications. The computational complexity is analyzed to ensure the suitability of the proposed encryption framework for real-time applications. The ViTAE module encodes an input image into a latent image. According to^64^, the complexity of the encoder of ViTAE is

$$\begin{aligned} T_E = O(L\times (N_pD^2 + N_p^2D)), \end{aligned}$$

where size of the input image is $P\times Q$, $N_p = ((P\times Q)/P_s)$ refers to total number of patches, $P_s$ is the patch size, *D* is the embedding dimension and *L* is the encoder depth. Here, $D \gg N_p$ in most of the cases so the term $N_pD^2$ dominates $N_p^2D$, which gives $T_E \approx O(LN_pD^2)$. The confusion and diffusion modules, RST and TCAI, of the encryption process undergo a single pass. Therefore, the complexity of RST is $T_{rst}=O(P \times Q)$, and that of TCAI is $T_{tcai}=O(P \times Q)$. The complexity to generate the chaotic sequences is $T_{seq} = O(P \times Q)$. Thus, the total time complexity of the proposed image encryption method is

$$\begin{aligned} \begin{array}{lll} T_{\text {total}} & =& T_{E} + T_{\text {rst}} + T_{\text {tcai}} + T_{\text {seq}}\\ & =& O(LN_pD^2)+ O(P \times Q) + O(P \times Q) + O(P \times Q)\\ & =& O(LN_pD^2+PQ) \end{array} \end{aligned}$$

In this experimental set up $P = Q = 256$, $P_s=32\times 32$, $L=6$, $D=2048$, and $N_p=64$. From theoretical complexity of the method, it is not always judge the usefulness of the method in real-time applications. Table 19 displays the encryption time required by the proposed method. For test images encryption consumes roughly 1.1867 seconds. Remarkably, the execution time of the suggested method encompasses both the time required to extract the latent image from the original image and the time required to encrypt the latent image. The comparison with SoA methods shown in Table 19 demonstrates that the suggested method achieves faster performance concerning systems’ architectures, highlighting the suggested method’s efficiency and practicality.

**Table 19:** Execution time analysis.

Method	Time (s)	System Configuration			
In^24^	139.0000	Intel Core i7-8700,	16 GB RAM,	CPU@ 3.2GHz,	GeForce RTX 2080Ti$\times$ 2
In^25^	1.8643	Intel Core i7,	8 GB RAM,	CPU@ 2.3 GHz	
Proposed	1.1867	Intel Core i5-10300H,	8 GB RAM,	CPU @ 2.5GHz	

## Conclusions and future work

This research introduces a color image encryption scheme that utilizes a 6D hyperchaotic system with a ViT-based autoencoder, providing a faster and secure approach for safeguarding sensitive image data. The proposed method utilizes the SHA3-512 algorithm to produce the hash value of the plain image and initialize the state variables of the 6DHCS. The 6DHCS exhibits chaotic behavior, which is validated through a rigorous analysis. The ViTAE model compresses the image into a compact latent representation. The encryption process of the latent image incorporates a combination of scrambling via chaotic sequences and Random Shuffle, followed by pixel-level diffusion using the Trifid Cipher. Experimental evaluations confirm the scheme’s resilience against various cryptography attacks, as well as its superior performance in terms of key space, key sensitivity, and computational speed. Though the proposed method performs well compared to the SoA methods, its major limitation is the loss of information, which restricts its applicability. The method lacks the resilience when noise is introduced into the latent encrypted representation.

Building on the promising performance of the current scheme, future work will focus on optimizing the encryption pipeline for hardware acceleration, particularly through the use of FPGAs and other parallel computing platforms^65,66^. These enhancements aim to reduce latency and improve throughput for real-time applications. Special attention will be given to tailoring the ViT-based compression and chaotic encryption stages for (i) efficient execution on resource-constrained devices such as IoT nodes and embedded systems, and (ii) robust handling of advanced cryptanalysis, adversarial reconstruction, and deep learning-based attacks.

## Data Availability

The datasets generated during and/or analysed during the current study are available from the corresponding authors on reasonable request.

## References

[1] Elkandoz, M. T. & Alexan, W. Image encryption based on a combination of multiple chaotic maps. *Multimed. Tools Appl.* **81**, 25497–25518 (2022).

[2] Ahmed, F. et al. A dna based colour image encryption scheme using a convolutional autoencoder. *ACM Trans. Multimed. Comput. Commun. Appl.* **19**, 1–21 (2023).

[3] El-Damak, D. et al. Fibonacci q-matrix, hyperchaos, and galois field () for augmented medical image encryption. *IEEE Access* **12**, 102718–102744 (2024).

[4] Hosny, K. M., Zaki, M. A., Lashin, N. A., Fouda, M. M. & Hamza, H. M. Multimedia security using encryption: A survey. *IEEE Access* **11**, 63027–63056 (2023).

[5] Rabah, K. Theory and implementation of data encryption standard: A review. *Infor. Tech. J.* **4**, 307–325 (2005).

[6] Abdullah, A. M. et al. Advanced encryption standard (aes) algorithm to encrypt and decrypt data. *Cryptogr. Netw. Secur.* **16**, 11 (2017).

[7] Zhou, X. & Tang, X. Research and implementation of rsa algorithm for encryption and decryption. In *Proceedings of 2011 6th international forum on strategic technology*, vol. 2, 1118–1121 (IEEE, 2011).

[8] Lai, Q., Hu, G., Erkan, U. & Toktas, A. A novel pixel-split image encryption scheme based on 2d salomon map. *Expert Syst. with Appl.* **213**, 118845 (2023).

[9] Zhang, Q. & Han, J. A novel color image encryption algorithm based on image hashing, 6d hyperchaotic and dna coding. *Multimed. Tools Appl.* **80**, 13841–13864 (2021).

[10] Moysis, L. et al. Exploiting circular shifts for efficient chaotic image encryption. *IEEE Access* **13**, 92997–93016 (2025).

[11] Devi, C. S. & Amirtharajan, R. A novel 2d mtmhm based key generation for enhanced security in medical image communication. *Sci. Rep.* **15**, 25411 (2025). 40659745 10.1038/s41598-025-10485-1 PMC12259849

[12] Anujaa, T. et al. A lightweight multi round confusion-diffusion cryptosystem for securing images using a modified 5d chaotic system. *Sci. Rep.* **15**, 31986 (2025). 40885745 10.1038/s41598-025-13290-y PMC12398579

[13] Alali, A. S., Ali, R., Jamil, M. K., Ali, J. & Gulraiz. Dynamic s-box construction using mordell elliptic curves over galois field and its applications in image encryption. *Mathematics*, **12**, 587 (2024).

[14] Ahmad Khan, N. et al. Unveiling a novel s-box strategy: The dynamic 3d scrambling approach. *Plos one* **20**, e0329024 (2025). 40892902 10.1371/journal.pone.0329024 PMC12404556

[15] Banga, A. et al. Where octagonal geometry meets chaos: A new s-box for advanced cryptographic systems. *PloS one* **20**, e0320457 (2025). 40489462 10.1371/journal.pone.0320457 PMC12148150

[16] Li, P., Pei, Y. & Li, J. A comprehensive survey on design and application of autoencoder in deep learning. *Appl. Soft. Comput.* **138**, 110176 (2023).

[17] He, K. et al. Masked autoencoders are scalable vision learners. In *Proceedings of the IEEE/CVF conference on computer vision and pattern recognition*, 16000–16009 (2022).

[18] Prabhakar, C. et al. Vit-ae++: improving vision transformer autoencoder for self-supervised medical image representations. In *Medical Imaging with Deep Learning*, 666–679 (PMLR, 2024).

[19] Fan, H., Cheng, S., de Nazelle, A. J. & Arcucci, R. Vitae-sl: A vision transformer-based autoencoder and spatial interpolation learner for field reconstruction. *Comput. Phys. Commun.* **308**, 109464 (2025).

[20] Maniyath, S. R. & Thanikaiselvan, V. An efficient image encryption using deep neural network and chaotic map. *Microprocess. Microsyst.* **77**, 103134 (2020).

[21] Fang, P., Liu, H. & Wu, C. A novel chaotic block image encryption algorithm based on deep convolutional generative adversarial networks. *IEEE Access* **9**, 18497–18517 (2020).

[22] Ding, Y. et al. Deepedn: A deep-learning-based image encryption and decryption network for internet of medical things. *IEEE Internet Things J.* **8**, 1504–1518 (2020).

[23] Man, Z., Li, J., Di, X., Sheng, Y. & Liu, Z. Double image encryption algorithm based on neural network and chaos. *Chaos, solitons & fractals* **152**, 111318 (2021).

[24] Wu, J. et al. Image encryption based on adversarial neural cryptography and sha controlled chaos. *J. Mod. Opt.* **68**, 409–418 (2021).

[25] Fang, P., Liu, H., Wu, C. & Liu, M. A secure chaotic block image encryption algorithm using generative adversarial networks and dna sequence coding. *Math. Probl. Eng.* **2021**, 6691547 (2021).

[26] Man, Z. et al. A novel image encryption algorithm based on least squares generative adversarial network random number generator. *Multimed. Tools Appl.* **80**, 27445–27469 (2021).

[27] Bao, Z., Xue, R. & Jin, Y. Image scrambling adversarial autoencoder based on the asymmetric encryption. *Multimed. Tools Appl.* **80**, 28265–28301 (2021).

[28] Ding, Y. et al. Deepkeygen: a deep learning-based stream cipher generator for medical image encryption and decryption. *IEEE Trans. Neural Netw. Learn. Syst.* **33**, 4915–4929 (2021). 10.1109/TNNLS.2021.3062754 33729956

[29] Wang, C. & Zhang, Y. A novel image encryption algorithm with deep neural network. *Signal Process.* **196**, 108536 (2022).

[30] Sang, Y., Sang, J. & Alam, M. S. Image encryption based on logistic chaotic systems and deep autoencoder. *Pattern Recognit. Lett.* **153**, 59–66 (2022).

[31] Zhou, S., Zhao, Z. & Wang, X. Novel chaotic colour image cryptosystem with deep learning. *Chaos, Solitons & Fractals* **161**, 112380 (2022).

[32] Erkan, U., Toktas, A., Enginoğlu, S., Akbacak, E. & Thanh, D. N. An image encryption scheme based on chaotic logarithmic map and key generation using deep cnn. *Multimed. Tools Appl.* **81**, 7365–7391 (2022).

[33] Wang, X. et al. A new v-net convolutional neural network based on four-dimensional hyperchaotic system for medical image encryption. *Secur. Commun. Networks* **2022**, 4260804 (2022).

[34] Sun, M., Yuan, J., Li, X., Liu, D. & Wei, X. Lvgg-ie: A novel lightweight vgg-based image encryption scheme. *Entropy* **26**, 1013 (2024). 39766642 10.3390/e26121013 PMC11727313

[35] Guo, Y., Chang, J., Zhang, Y., Zhang, J. & Ma, S. Image encryption and compression based on reversed diffusion model. In *2024 Picture Coding Symposium (PCS)*, 1–5 (IEEE, 2024).

[36] Kadhim, Q. & Al-Jawher, W. A. M. A new multiple-chaos image encryption algorithm based on block compressive sensing, swin transformer, and wild horse optimization. *Multidiscip. Sci. J.* **7**, 2025012–2025012 (2025).

[37] Huang, Y. et al. An image encryption framework based on chaotic sequence combined with deep learning. *Physica Scripta* **100**(11), 115208 (2025).

[38] Alexan, W. et al. Secure communication of military reconnaissance images over uav-assisted relay networks. *IEEE Access* **12**, 78589–78610 (2024).

[39] Yu, W. et al. Design of a new seven-dimensional hyperchaotic circuit and its application in secure communication. *IEEE Access* **7**, 125586–125608 (2019).

[40] Khalaf, A. J. M. et al. Extreme multi-stability analysis of a novel 5d chaotic system with hidden attractors, line equilibrium, permutation entropy and its secure communication scheme. *Eur. Phys. J. Special Top.* **229**, 1175–1188 (2020).

[41] Cui, N. & Li, J. A new 4d hyperchaotic system and its control. *Aims Math* **8**, 905–923 (2023).

[42] Vaidyanathan, S. Hyperchaos, adaptive control and synchronization of a novel 4-d hyperchaotic system with two quadratic nonlinearities. *Arch. Control. Sci.* **26**, 471–495 (2016).

[43] Kouadra, I. et al. New composite chaotic map applied to an image encryption scheme in cybersecurity applications. *IEEE Access* **13**, 70676–70690 (2025).

[44] Gottwald, G. A. & Melbourne, I. A new test for chaos in deterministic systems. *Proc. Royal Soc. London. Ser. A: Math.Phys. Eng. Sci.* **460**, 603–611 (2004).

[45] Gottwald, G. A. & Melbourne, I. On the implementation of the 0–1 test for chaos. *SIAM J. on Appl. Dyn. Syst.* **8**, 129–145 (2009).

[46] Jasim, B. H., Mjily, A. H. & Al-Aaragee, A. M. J. A novel 4 dimensional hyperchaotic system with its control, synchronization and implementation. *Int. J. Electr. Comput. Eng.* **11**, 2974 (2021).

[47] Rukhin, A. et al. A statistical test suite for random and pseudorandom number generators for cryptographic applications (2001).

[48] Choi, Y., Uh, Y., Yoo, J. & Ha, J.-W. Stargan v2: Diverse image synthesis for multiple domains. In *Proceedings of the IEEE Conference on Computer Vision and Pattern Recognition* (2020).

[49] Wang, Z., Bovik, A. C., Sheikh, H. R. & Simoncelli, E. P. Image quality assessment: from error visibility to structural similarity. *IEEE Trans. Image Process.* **13**, 600–612 (2004). 15376593 10.1109/tip.2003.819861

[50] Wang, Z., Simoncelli, E. P. & Bovik, A. C. Multiscale structural similarity for image quality assessment. In *The thrity-seventh asilomar conference on signals, systems & computers, 2003*, vol. 2, 1398–1402 (IEEE, 2003).

[51] Zhang, L., Zhang, L., Mou, X. & Zhang, D. Fsim: A feature similarity index for image quality assessment. *IEEE Trans. Image Process.* **20**, 2378–2386 (2011). 21292594 10.1109/TIP.2011.2109730

[52] Sheikh, H. R. & Bovik, A. C. Image information and visual quality. *IEEE Trans. Image Process.* **15**, 430–444 (2006). 16479813 10.1109/tip.2005.859378

[53] Fisher, R. A. & Yates, F. *Statistical tables for biological, agricultural and medical research* (Hafner Publishing Company, 1953).

[54] Knuth, D. E. *The Art of Computer Programming: Seminumerical Algorithms, Volume 2* (Addison-Wesley Professional, 2014).

[55] Delastelle, F. *Traité élémentaire de cryptographie: mathematiques appliquées* (Gauthier-Villars, 1902).

[56] SIPI Image Database. https://sipi.usc.edu/database/.

[57] Alvarez, G. & Li, S. Cryptographic requirements for chaotic secure communications. *arXiv preprint* arXiv:nlin/0311039 (2003).

[58] Toktas, A., Erkan, U., Gao, S. & Pak, C. A robust bit-level image encryption based on bessel map. *Appl. Math. Comput.* **462**, 128340 (2024).

[59] Wu, Y. et al. Local shannon entropy measure with statistical tests for image randomness. *Inf. Sci.* **222**, 323–342 (2013).

[60] Maity, A. & Dhara, B. C. Image encryption utilizing 5d hyperchaos, wavelet lifting scheme, and burrows–wheeler transform. *Arab. J. for Sci. Eng.* **50**, 19403–19419 (2025).

[61] Iqbal, N. et al. Utilizing the nth root of numbers for novel random data calculus and its applications in network security and image encryption. *Expert Syst. with Appl.* **265**, 125992 (2025).

[62] Maiti, C., Dhara, B. C., Umer, S. & Asari, V. An efficient and secure method of plaintext-based image encryption using fibonacci and tribonacci transformations. *IEEE Access* **11**, 48421–48440 (2023).

[63] Npcr and uaci randomness tests for image encryption. *Cyber journals: multidisciplinary journals in science and technology, Journal of Selected Areas in Telecommunications (JSAT)* **1**, 31–38 (2011).

[64] Vaswani, A. et al. Attention is all you need. *Adv. Neural Inf. Process. Syst.* **30** (2017).

[65] Alexan, W. et al. Triple layer rgb image encryption algorithm utilizing three hyperchaotic systems and its fpga implementation. *IEEE Access* **12**, 118339–118361 (2024).

[66] Alexan, W., Hosny, K. & Gabr, M. A new fast multiple color image encryption algorithm. *Clust. Comput.* **28**, 1–34 (2025).

